# Downregulation of SNRPG induces cell cycle arrest and sensitizes human glioblastoma cells to temozolomide by targeting Myc through a p53-dependent signaling pathway

**DOI:** 10.20892/j.issn.2095-3941.2019.0164

**Published:** 2020-02-15

**Authors:** Yulong Lan, Jiacheng Lou, Jiliang Hu, Zhikuan Yu, Wen Lyu, Bo Zhang

**Affiliations:** ^1^Department of Neurosurgery, Shenzhen People’s Hospital, Second Clinical Medical College of Jinan University, The First Affiliated Hospital of Southern University of Science and Technology, Shenzhen 518020, China; ^2^Department of Neurosurgery, The Second Affiliated Hospital of Dalian Medical University, Dalian 116023, China

**Keywords:** SNRPG, glioblastoma, cell cycle, temozolomide, therapy

## Abstract

**Objective:** Temozolomide (TMZ) is commonly used for glioblastoma multiforme (GBM) chemotherapy. However, drug resistance limits its therapeutic effect in GBM treatment. RNA-binding proteins (RBPs) have vital roles in posttranscriptional events. While disturbance of RBP-RNA network activity is potentially associated with cancer development, the precise mechanisms are not fully known. The *SNRPG* gene, encoding small nuclear ribonucleoprotein polypeptide G, was recently found to be related to cancer incidence, but its exact function has yet to be elucidated.

**Methods:** *SNRPG* knockdown was achieved via short hairpin RNAs. Gene expression profiling and Western blot analyses were used to identify potential glioma cell growth signaling pathways affected by *SNRPG*. Xenograft tumors were examined to determine the carcinogenic effects of *SNRPG* on glioma tissues.

**Results:** The *SNRPG*-mediated inhibitory effect on glioma cells might be due to the targeted prevention of Myc and p53. In addition, the effects of *SNRPG* loss on p53 levels and cell cycle progression were found to be Myc-dependent. Furthermore, *SNRPG* was increased in TMZ-resistant GBM cells, and downregulation of *SNRPG* potentially sensitized resistant cells to TMZ, suggesting that *SNRPG* deficiency decreases the chemoresistance of GBM cells to TMZ via the p53 signaling pathway. Our data confirmed that *SNRPG* suppression sensitizes GBM cells to TMZ by targeting Myc via the p53 signaling cascade.

**Conclusions:** These results indicated that *SNRPG* is a probable molecular target of GBM and suggested that suppressing *SNRPG* in resistant GBM cells might be a substantially beneficial method for overcoming essential drug resistance.

## Introduction

Glioblastoma multiforme (GBM) is the most commonly occurring malignant form of brain cancer in adults. The median overall survival of GBM patients remains between 6–15 months, with a 5 year survival rate from the date of diagnosis of less than 5%^1,2^. Without treatment, the majority of patients with GBM survive for only a few months^3^, and new therapeutic approaches are urgently needed. The SNRPG protein is a core element of U1, U2, U4, and U5 small nuclear ribonucleoprotein (snRNP) complexes, which are the building blocks (or originators) of the spliceosome, and might also be a component of the U7 snRNP complex, which is involved in processing of the 3' ends of histone transcripts. Numerous transcript alternates encoding various isoforms of *SNRPG* have been identified (http://www.genecards.org/), and the molecular mechanisms underlying the roles of *SNRPG* in GBM need to be clarified.

Myc can mediate a transcriptional program encompassing cell growth, metabolism, the cell cycle, and survival in cancer cells^4,5^. Substantial effort has been devoted to targeting Myc for cancer therapy, and Myc inhibition appears to be of significant therapeutic value for cancers expressing high levels of Myc^6^. Moreover, Myc expression correlates with the glioma grade^7^, and approximately 60%–80% of GBMs exhibit increased Myc levels^8^. Importantly, preclinical studies have validated Myc inhibition as an effective therapeutic strategy for human gliomas^9^, and the identification of new protein-coding genes and the development of novel compounds to pharmacologically target Myc-driven cancers are key research goals. The p53 (also known as TP53) protein is a well-known cancer suppressor with pleiotropic roles, as it regulates transcription by binding to exact DNA sequences^10–12^ and to other cellular proteins, such as Mdm2, TBP, and Gadd45^13–15^. The p53 also participates in DNA replication^16^ and restoration procedures^17^. Interestingly, in numerous mouse models of Myc-driven tumors, tumor deterioration *via* Myc repression is hindered by simultaneous suppression of the TP53 protein, highlighting the relevance of an intact p53 pathway for treating cancer by targeting Myc^18–20^.

Temozolomide (TMZ) chemotherapy shows remarkable therapeutic enhancement by extending tumor control as well as patient survival in newly detected GBM^21^. However, the effective rate of TMZ is only 35%^22^, and overcoming chemoresistance is thus essential for enhancing the survival rate of GBM patients^23^. Intriguingly, p53 has been substantially associated with the efficacy of TMZ treatment for GBM, and contradictory results regarding the clinically significant influence of the p53 status on TMZ resistance have been reported^24^. Various studies have shown either an enhanced ability of TMZ to prevent cell viability when p53wt is functionally repressed^25,26^ or a sensitization of cells to medications when p53wt is efficient^27,28^. Nuclear overexpression of p53 generally reflects a marker of mutation, and numerous studies have indicated that expression of p53 is 90% associated with its mutation^29^. In cells containing mutant p53, TMZ causes temporary cell cycle arrest in addition to cell death through apoptosis or mitotic catastrophe^30^ along with attenuated DNA repair^31^. Thus, the activation of p53 may contribute to the efficacy of TMZ for treating GBM.

Because *SNRPG* is a favorable candidate based on gene screening, we examined its roles in GBM occurrence and tumor development as well as TMZ resistance. Interestingly, the association between *SNRPG* and Myc- and p53-mediated cell cycle signaling required further elucidation. In this study, we performed experiments both *in vivo* and *in vitro* to detect the exact mechanisms by which *SNRPG* promotes GBM. Targeting the *SNRPG*-driven p53 signaling axis is a potential therapeutic strategy for effective GBM treatment and for overcoming chemoresistance.

## Materials and methods

All experiments were performed in accordance with regular institutional biosecurity and institutional safety processes. Each experimental workplace included a biosafety level 1 (BSL-1) facility, and all individuals involved were committed to using BSL-1 techniques.

### Cancer patient samples

Experiments on human subjects followed the Helsinki Declaration^32^ and were approved by the Ethics Committee of Dalian Medical University (reference number: KY2019-01). When donor material/samples were obtained from deceased patients, written informed consent was obtained from the patients’ family members. Materials were harvested from 15 fresh tissue samples obtained from GBM patients who were admitted to the Second Affiliated Hospital of Dalian Medical University from September 2014 to December 2016 (**Supplementary Table S1**). Normal brain tissues were acquired from the Institute of Biochemistry and Cell Biology (Shanghai, China).

### Cell culture

The authenticities of the human U87 MG, U251, LN229, and A172 cells were verified based on their genomic short tandem repeat (STR) profiles by GeneChem Biotechnology Co., Ltd. (Shanghai, China), while the U118 cell line was verified based on its genomic STR profiles by Shanghai Zhong Qiao Xin Zhou Biotechnology Co., Ltd. (Shanghai, China). The cell lines were determined to be free of mycoplasma using the Mycoplasma Detection Kit-Quick Test (Biotool Biotechnology, Shanghai, China). The human U87MG (ATCC® HTB-14), LN229 (ATCC® CRL-2611), A172 (ATCC® CRL-1620), and U118 (ATCC® HTB-15) cell lines were acquired from the American Type Culture Collection (ATCC, Old Town, Manassas, VA, USA). The U251 cell line was obtained from the Type Culture Collection of the Chinese Academy of Sciences (Shanghai, China). The human astrocytes were obtained from ScienceCell (San Diego, CA, USA). Normal brain tissues were acquired from the Institute of Biochemistry and Cell Biology (Shanghai, China). Cells were preserved in Dulbecco’s Modified Eagle’s Medium supplemented with 10% fetal bovine serum. All cell cultures were maintained at 37 °C in a humidified environment containing 5% CO_2_. The TMZ-resistant GBM cell lines were established as described below. U87 cells (1 × 10^5^/mL) were first incubated for 24 h and then treated with an initial concentration of TMZ (5 µM), and the medium containing TMZ was changed every 2–3 days. After application of the initial dosage for 2 weeks, the dosage was doubled, and each dosage was continued for 14 days; the ultimate dose reached 400 µM. The U87 drug-resistant cell line was labeled U87/TMZ. The technique used to induce the U251 drug-resistant cell line was similar to that used for the U87/TMZ line.

### SNRPG knockdown by shRNA

Cells were plated in a 6-well plate (~5 × 10^4^ cells/well) and then incubated at 37 °C with 5% CO_2_ until reaching a confluence of ~30%, after which the cells were transfected. Lentiviral vectors were purchased from Shanghai GeneChem Company Ltd. (Shanghai, China). A nonsilencing shRNA (5'-GCCTAACTGTGTCAGAAGGAA-3') was used as the negative control (shCtrl). The shRNA sequence targeting the *SNRPG* gene was 5'-TGGACAACAGAACAATATT-3'.

### Cell viability assay

Cell viability was measured by the [3-(4,5-dimethylthiazol-2-yl)-2,5-diphenyltetrazolium bromide] (MTT) assay (Roche Diagnostics, Santa Clara, CA, USA). Cell lines were plated at 6 × 10^3^ cells/well in 96-well plates and allowed to adhere for more than 5 days after transfection. The cell growth was then evaluated using optical density values.

### Celigo assay

U87MG or U251 cells in the logarithmic growth phase were processed with trypsin, resuspended in standard medium, and then plated in 96-well plates (2,000 cells/well). The amount of green fluorescent protein-positive cells was calculated on five consecutive days using a Cellomics Array Scan High-Content Screening Reader (Olympus Corporation, Tokyo, Japan).

### Cell cycle analysis

First, the cells were trypsinized into single cells, collected, washed with phosphate-buffered saline (PBS) and suspended in a staining buffer [10 µg/mL propidium iodide (PI), 0.5% Tween 20, 0.1% RNase in PBS]. The stained cells were examined using an Accuri C6 fluorescence-activated cell sorter (FACS) (Genetimes Technology Inc., Shanghai, China).

### Apoptosis analysis

Apoptosis was measured by FACS using an annexin V-fluorescein isothiocyanate (FITC) apoptosis detection kit (Nanjing KeyGEN Biotech. Co. Ltd., China). In brief, the cells were plated in 6-well plates, collected, washed once with cold PBS, and immediately stained with FITC-labeled annexin V and PI. These stained cells were examined using the FACS Accuri C6 instrument (Genetimes Technology Inc., Shanghai, China).

### High resolution O^6^-methylguanine-DNA methyltransferase gene (MGMT) methylation analysis

Bisulfite samples were analyzed using high sensitive SYBR® Green (KapaBiosystems, Wilmington, MA, USA) at the Center for Genomics and Oncological Research, University of Granada (Granada, Spain). The reaction was performed on an Eco Real-Time PCR System (Illumina, San Diego, CA, USA), and data were evaluated by Eco Real-Time PCR System, version 4.0 software (Illumina). Methylated EpiTect Control DNA, both methylated and unmethylated (Qiagen, Madrid, Spain), was used to construct the methylation curve at methylated-unmethylated ratios of 0, 0.25, 0.5, 0.75, and 1. A pair of primers capable of amplifying a precise region was used to analyze both the samples and the methylation curve.

### Western blot analysis

Cells were lysed, and the proteins were separated on 7.5%–12% sodium dodecyl sulfate-polyacrylamide gel electrophoresis mini gels and then transferred to a polyvinylidene fluoride membrane. The blots were incubated with appropriate antibodies, and the protein bands were identified using enhanced chemiluminescence. Analogous experiments were performed at least 3 times. The total protein concentration was identified using a bicinchoninic acid protein assay kit. Primary antibodies against glyceraldehyde 3-phosphate dehydrogenase (GAPDH) were acquired from Santa Cruz Biotechnology (Dallas, TX, USA), and an anti-CCNB1 antibody was purchased from Proteintech (Hubei, China). Antibodies against Myc, p53, CDK2, P-gp, and GST-π were purchased from Abcam, Cambridge, UK. Primary antibodies against SNRPG and β-actin were acquired from Sigma-Aldrich (Shanghai, China) and Santa Cruz Biotechnology, respectively.

### Quantitative real-time reverse transcription polymerase chain reaction (RT-qPCR)

Total RNA was extracted from glioma and adjacent normal tissues using TRIzol reagent according to the manufacturer’s instructions (TaKaRa Bio, Beijing, China). The cDNA was reverse transcribed using the PrimeScript RT Reagent Kit (TaKaRa Bio) according to the manufacturer’s protocol. The qPCR was performed according to the kit instructions (TaKaRa Bio) using the Mx3005P Real-Time PCR System (Agilent, Santa Clara, CA, USA). GAPDH RNA levels were used to normalize the relative mRNA expression. The data were evaluated by the 2^−ΔΔCT^ method.

### Microarray gene expression analysis

U87MG cells were transfected with shSNRPG; total RNA was extracted, and 50–500 ng was used to create biotin-modified amplified RNA (aRNA) using a GeneChip 3' IVT Express Kit (Affymetrix, Santa Clara, CA, USA). Reverse transcription was performed using a T7 oligo (dT) primer. To generate numerous biotin-modified aRNA copies, a First-Strand IVT Labeling Master Mix (Thermo Fisher Scientific, Waltham, MA, USA) was used, and the aRNA was then purified and quantified. Following fragmentation, the aRNA was hybridized to the GeneChip PrimeView Human Expression Array (Affymetrix). Following hybridization, the chips were stained with phycoerythrin and washed using the Genechip Fluidics Station 450 (Thermo Fisher Scientific). The Genechip Array Scanner 3000 7G (Thermo Fisher Scientific) was used to scan and analyze the microarray signals.

Gene signatures were used to compare shCtrl to shSNRPG. To recognized pathways that were generally deregulated in U87MG-shSNRPG cells, and enhancement of differentially expressed gene signatures in human pathways were assessed using gene set enrichment analysis (GSEA); the pathways were collected in the H and C2 curated gene sets using 1,000 gene label permutations (gene sets). Significantly augmented gene sets, well-defined by *P* < 0.05, were compared among the shCtrl and shSNRPG gene signatures.

### Lentivirus construction, production, and infection

The *SNRPG* lentivirus plasmid was constructed, produced, and used to infect glioma cells as described in a previous publication^33^. At 48 h after infection, the virus-infected cells were cultured in medium containing 2.5 µg/mL puromycin (Sigma-Aldrich, St. Louis, MO, USA) for selection. The surviving cells were used for subsequent experiments. The *SNRPG*-overexpressing cells were named Lenti-SNRPG; the corresponding control cells were named Lenti-Vector.

### Confocal immunofluorescence

Immunofluorescence staining was performed in cells cultured on chamber slides. The cells were washed with PBS and then fixed with 4% paraformaldehyde for 10 min at room temperature. The samples were permeabilized with 0.2% Triton X-100 for 5 min and then blocked with 10% bovine serum albumin in PBS for 30 min. Antibodies against Myc and p53 in 1% blocking solution were added to the sample and incubated overnight (4 °C). After washing the samples with PBS 3 times, fluorescein isothiocyanate- and rhodamine-conjugated secondary antibodies were added to a 1% blocking solution, and the mixtures were incubated for 1 h. The stained samples were mounted using 4',6-diamidino-2-phenylindole-containing Vectashield solution (Vector Laboratories Inc., Burlingame, CA, USA) to counterstain the nuclei. After 5 additional 5 min washes, the samples were examined using a Leica DM 14000B confocal microscope (Leica, Wetzlar, Germany).

### Protein-ligand docking

The molecular docking simulations were conducted using Molecular Operating Environment (MOE) software, version 2018.01 (Chemical Computing Group Inc., Montreal, Canada). The two-dimensional structure of the small molecule, TMZ, was drawn using ChemBioDraw 2014 (PerkinElmer, Waltham, MA, USA) and converted into three-dimensional (3D) conformations using energy minimization. The 3D structure of the SNRPG protein was downloaded from the Research Collaboratory for Structural Bioinformatics Protein Data Bank (PDB, code 5XJL; https://www.rcsb.org/) and prepared using the QuickPrep module (MOE; Chemical Computing Group Inc.). The protonation states and orientations of the hydrogens were optimized by LigX (MOE; Chemical Computing Group Inc.) at a pH of 7 and a temperature of 300 K. The docking process was performed under the AMBER10/EHT force field (MOE; Chemical Computing Group Inc.) with an internal dielectric constant of 1, an external dielectric constant of 80, and an implicit solvation model of the reaction field. The Triangle Matcher (MOE; Chemical Computing Group Inc.) algorithm was used for the initial placement of 1,000 returned conformations, and the top 100 conformations ranked by the London dG (MOE; Chemical Computing Group Inc.) scoring function were further refined through energy minimization and then rescored using the Generalized Born/Volume Integral (MOE; Chemical Computing Group Inc.) solvation energy, which was a more accurate implicit solvent model scoring function. The induced-fit protocol was applied for energy minimization in which the ligand was fully flexible during the conformation sampling process, and the side chains of the receptor were also allowed to move. Finally, the 20 top-ranked poses were retained, and the most representative pose was retrieved by visual inspection for further analysis.

### Animal studies

To evaluate the effect of *SNRPG* knockdown in the human U87MG glioblastoma mouse model (female nu/nu mice aged 4–6 weeks), U87MG cells (2 × 10^6^ in 100 mL of PBS) were subcutaneously injected using a 27-gauge needle near the axillary fossa. These mice were randomly divided into 2 groups with 10 mice each. Tumors were measured daily with calipers, and the tumor volumes were calculated according to the formula V = 1/2 (width^2^ × length). The body weights were also recorded.

The cellular bioluminescence of each group was assayed by adding 5 µL of Pierce D-luciferin (Thermo Fisher Scientific, Rockford, IL, USA) to the cell culture medium and incubating the mixture for 20 min. In total, 100 µL of culture medium was mixed with 100 µL of 1% agarose in a cylindrical glass tube.

To monitor tumor growth, bioluminescence imaging with the IVIS Spectrum system (Caliper, Xenogen, Alameda, CA, USA) was initiated approximately 28 days after tumor implantation. The tumor-bearing mice were anesthetized (isoflurane/O_2_ in an induction chamber; isoflurane from Baxter International Inc., Deerfield, IL, USA), and a solution of D-luciferin (120 mg/kg in PBS in a total volume of 80 mL; Biosynthesis, Naperville, IL, USA) was administered subcutaneously to the neck region. Anesthesia was maintained with isoflurane (2%) in oxygen (1 dm^3^/min). Beginning 5 min after luciferin injection, the images were acquired at various exposure times (1, 5, 30, and 60 s). Data were quantified with Living Imaging software (PerkinElmer) using absolute photon counts (photons/s) in a region of interest that was manually drawn to outline the bioluminescence image signals.

On day 30 after tumor cell inoculation, all experimental mice were sacrificed with ether anesthesia, and the total weight of the tumors in each mouse was measured. The tumor tissues were harvested, freshly fixed with 10% neutral formalin, desiccated and embedded in paraffin. Four µm sections were stained with hematoxylin and eosin (H&E) and rabbit anti-proliferating cell nuclear antigen (PCNA; 1:100) and examined using a light microscope and a DM 4000B fluorescence microscope equipped with a digital camera (Leica).

All animals were purchased from the Experimental Animal Center, Dalian Medical University [certificate of conformity: no. SYXK (Liao) 2018-0007 and SCXK (Liao) 2018-0003]. This study was conducted in accordance with recommendations of the National Institutes of Health Guide for Care and Use of Laboratory Animals (Publication No. 85-23, revised 1985). All animal maintenance and procedures were performed in strict accordance with the recommendations established by the guidelines in the U.S. National Institutes of Health Guide for the Care and Use of Laboratory Animals.

### Statistical analysis

Statistical analyses were performed with GraphPad Prism and GraphPad InStat software (GraphPad Software, La Jolla, CA, USA). Student’s *t*-test was used to compare the values for the test and control samples *in vitro* and *in vivo*. SPSS statistical software for Windows, version 17.0 (SPSS, Chicago, IL, USA) was used for all statistical analyses. *P* < 0.05 was considered a statistically significant difference, ^*^P < 0.05; ^**^P < 0.01; ^***^P < 0.001.

## Results

### SNRPG expression is upregulated in human GBM tissues

**Figure 1:**
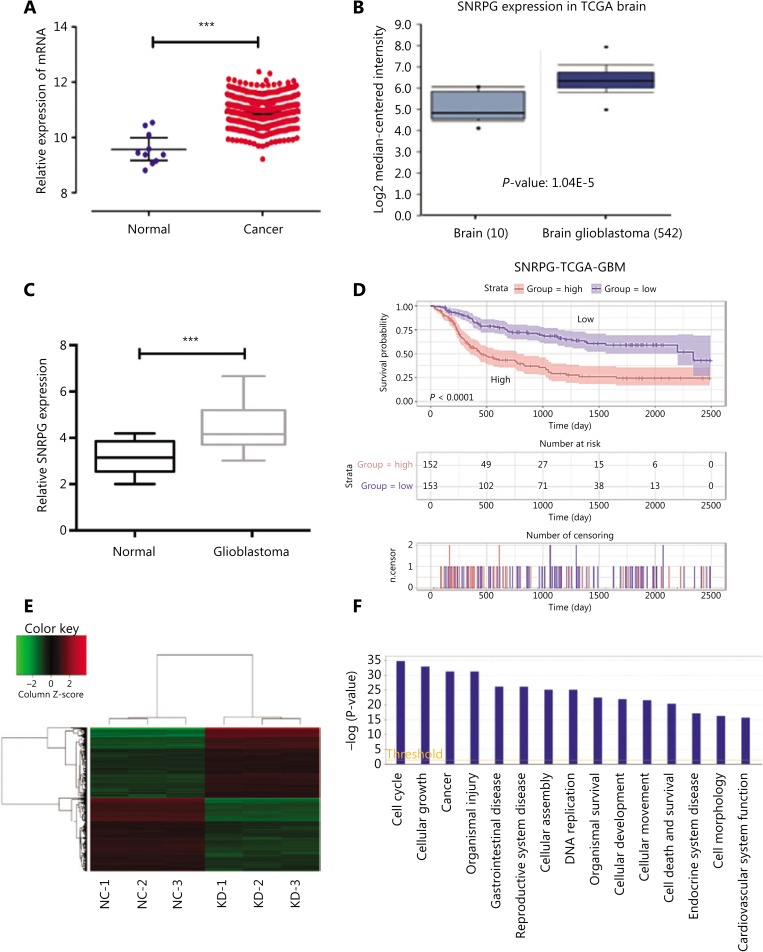
The involvement of *SNRPG* in human glioblastoma multiforme (GBM). (A) The correlation of *SNRPG* RNA expression levels with the incidence of GBM was examined *via* the *t*-test using the R programming language. The selection criteria were a *P* < 0.05 and |logFC| ≥ 1 (with 2 as the base). FC = 2.46, *P* = 3.86E-16. (B) Using Oncomine (https://www.oncomine.org/resource/login.html), the *SNRPG* expression in GBM tissues (*n* = 542) was compared with that in normal tissues (*n* = 10) in the “TCGA brain” of the TCGA dataset, revealing that *SNRPG* was significantly upregulated in GBM tissue compared with that in normal tissue. (C) *SNRPG* expression was examined in 15 GBM tissues and nontumor tissues by RT-qPCR, revealing significantly increased expression in GBM tissues compared with that in the normal tissue samples. Error bars, standard error. ^***^P < 0.001. (D) Kaplan-Meier survival analyses of GBM with different levels of SNRPG using the TCGA dataset and the R programming language. (E-F) Gene expression profile analysis revealed the downstream targets affected by *SNRPG*. (E) The heat map shows the differentially expressed genes in all samples. (F) The histogram of disease and function shows the enrichment of differential genes in disease and their functional classifications.

*SNRPG* expression in GBM tissues was compared with that in healthy tissues according to The Cancer Genome Atlas (TCGA) and found to be significantly upregulated by using the R programming language (**Figure 1A**). We also re-evaluated *SNRPG* expression in GBM and healthy tissues in other publicly available data on the Oncomine website (https://www.oncomine.org/), and found that *SNRPG* expression was also augmented in GBM according to the Oncomine dataset (**Figure 1B**). To confirm this possibility, RT-qPCR was performed to determine *SNRPG* expression in 15 pairs of GBM tissues as well as in normal brain tissues (**Figure 1C**). The results suggested increased expression of SNRPG in GBM tissues compared with that in normal tissue samples (*P* < 0.001). In addition, the prognostic signature of the *SNRPG* gene was also confirmed (**Figure 1D**). Overall, the results suggested that GBM progression was always accompanied by upregulation of SNRPG.

To further elucidate the roles of *SNRPG* in tumors, we compared the gene expression in U87MG-shCtrl and U87MG-shSNRPG cells by microarray analysis. Differentially expressed genes with at least a 1.5-fold variation were recognized. First, a heat map was used to illustrate the aggregation of all samples and genes with differential expression (**Figure 1E**). A histogram of disease and function helped to illustrate the enrichment of differentially expressed genes in disease and to classify their functions (**Figure 1F**), which showed that the level of SNRPG expression could be associated with the cell cycle, cell growth, and more importantly, the incidence of cancer.

### Functional loss of SNRPG inhibits GBM cell proliferation ***in vitro***

**Figure 2:**
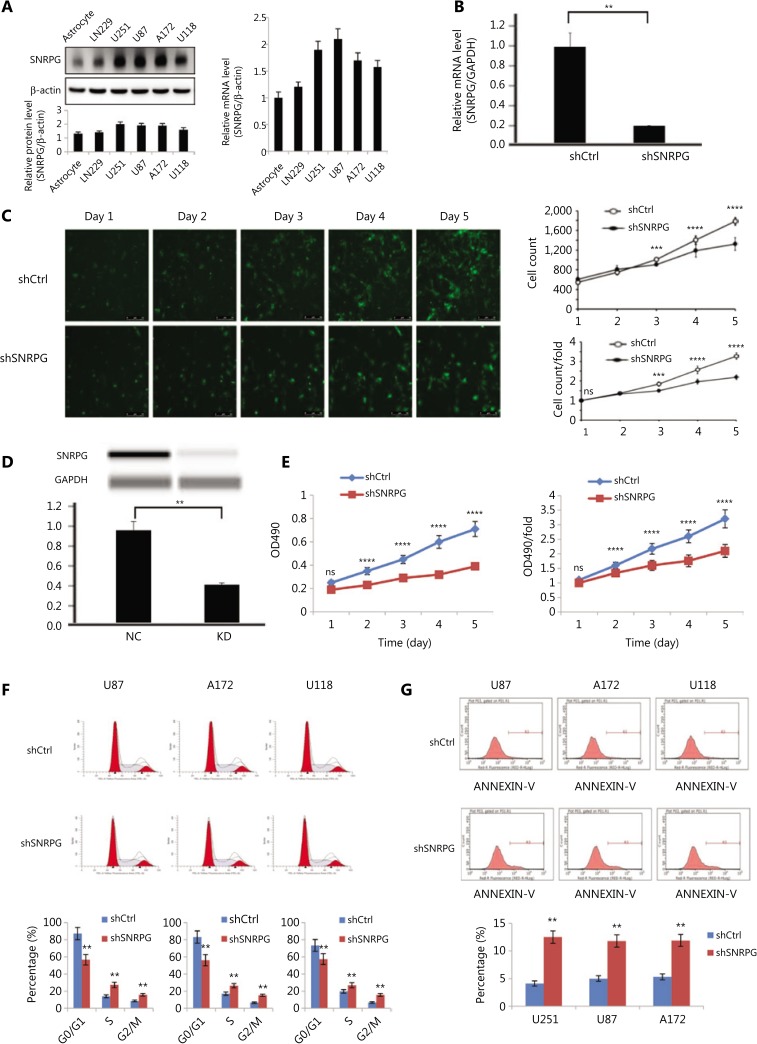
Effects of SNRPG knockdown on cell proliferation. (A) Expression of SNRPG in normal astrocyte and glioma cell lines detected by Western blot and RT-qPCR. The RT-qPCR results indicated that all glioblastoma cell lines exhibited high expression of SNRPG (mean ± SD, *n* = 3). The U87MG and U251 cell lines were selected for further study to investigate the biological role of SNRPG in GBM cells. (B) SNRPG was knocked down using shRNA (mean ± SD, *n* =3, **P < 0.01). (C) The Celigo assay was performed to detect cell viability over 5 days after transfection. The colony numbers of U87MG cells transfected with shCtrl were larger than those of U87MG cells transfected with shSNRPG (mean ± SD, *n* = 3, ns, not significant; **P < 0.01; ***P < 0.001; ****P < 0.0001). Bar = 50 μm. (D) Simple Western lane view with results automatically generated by system software. The downregulated expression of SNRPG by shSNRPG in U87MG cells was further confirmed *via* a simple Western size assay (**P < 0.01). (E) The MTT assay showed that SNRPG knockdown significantly decreased the proliferation of U87MG cells compared with that of the control cells. The optical density values were measured on the indicated days (mean ± SD, *n* = 3, ns, not significant; ****P < 0.0001). (F) Flow cytometry analysis was performed to further examine the effect of SNRPG on the proliferation of glioblastoma multiforme cells by altering cell cycle progression. Error bars, standard error. **P < 0.01. (G) To further determine the physiological role of SNRPG in cell growth, U87MG, A172, and U118 cells were transfected with shSNRPG. After 48 h, cell apoptosis was examined by flow cytometry analysis. Graphs show the mean percentages of three biological replicates. Error bars, standard error. **P < 0.01.

First, using Western blot (**Figure 2A, left panel**) and RT-qPCR (**Figure 2A, right panel**), we assayed the expression of *SNRPG* in normal brain tissues and in the human U251, A172, U87, LN229, and U118 cell lines. To investigate the effects of *SNRPG* in GBM cells, we used the U87 and U251 cell lines because they exhibited the highest relative expression of *SNRPG* (**Figure 2A**). *SNRPG* knockdown was achieved by shRNA (**Figure 2B**), and the Celigo assay was performed to identify cell viability. **Figure 2C** shows that the survival rate of U87MG cells transfected with shCtrl was higher than that of U87MG cells transfected with shSNRPG (^**^P < 0.01; ^***^P < 0.001; ^****^P < 0.0001) (**Figure 2C**). Western blot assays confirmed that SNRPG protein expression was downregulated in SNRPG-silenced U87MG cells compared to that in shCtrl-transduced cells (**Figure 2D**). The MTT assay also revealed that silencing *SNRPG* expression increased U87MG cell production compared to that in control cells (^****^P < 0.0001) (**Figure 2E**), indicating that functional loss of SNRPG inhibited GBM cell proliferation. Flow cytometry was performed to determine whether *SNRPG* affected the proliferation of GBM cells by altering cell cycle progression. The transfection of GBM cells with shSNRPG blocked cell cycle development via inducing G2/M cell cycle arrest (^**^P < 0.01) (**Figure 2F**). Moreover, to define the physiological part of SNRPG in apoptosis, U87MG, A172, and U118 cells were transfected with shSNRPG, and apoptosis was investigated by flow cytometry after 48 h, to show that transfection with shSNRPG significantly promoted apoptosis (^**^P < 0.01) (**Figure 2G**). The experimental results obtained in another GBM cell line, U251, were consistent with those obtained using U87MG, A172, and U118 cells (**Supplementary Figure S1**). In addition, experiments using GBM neurospheres with serum-free medium also showed that SNRPG inhibition suppressed the proliferation and invasiveness of neurospheres from GBM patients (**Supplementary Figure S2**). Furthermore, the expression levels of SNRPG in samples from primary and recurrent GBM patients and the possible correlation between SNRPG expression and the MGMT methylation status were also determined (**Supplementary Figure S2**). The role of SNRPG in the context of mismatch repair was also analyzed, showing that SNRPG inhibition increased the protein expression of the mismatch repair (MMR) protein MLH1, whose decreased expression could be a well-established mechanism of TMZ resistance^34^ (**Supplementary Figure S2**). Together, these results indicated that functional SNRPG damage inhibited GBM cell propagation, blocked cell cycle development, and promoted apoptosis *in vitro*.

### Functional loss of SNRPG inhibits GBM tumorigenesis *in vivo*

**Figure 3:**
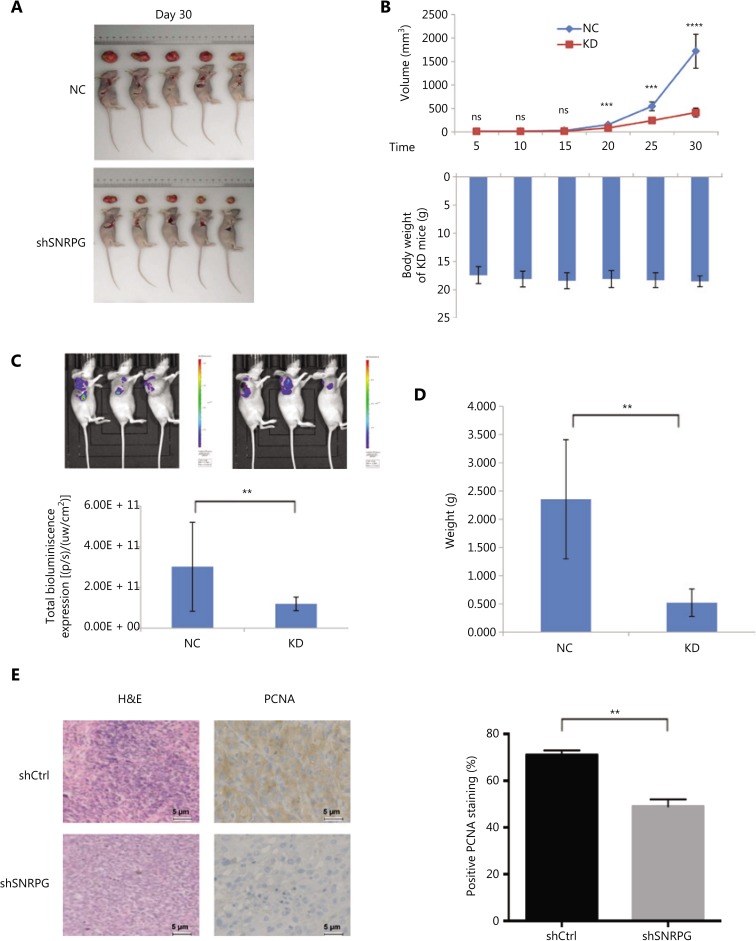
Effects of *SNRPG* knockdown on xenograft tumorigenicity *in vivo* in nude mice. U87MG cells were injected into the flanks of the nude mice. (A) Tumor growth was measured on the indicated days. (B) Tumor volumes (upper panel) and body weights (lower panel) were measured on the indicated days (mean ± SD, *n* = 10, ns, not significant; ^***^P < 0.001; ^****^P < 0.0001 *vs.* the shCtrl group on the indicated days). (C) Representative bioluminescence images of shCtrl- and shSNRPG-infected U87MG cells injected into the flanks of nude mice. Mice were imaged 28 days after implantation (*n* = 10 in the shCtrl group and *n* = 20 in the shSNRPG group). *In vivo* imaging detection clearly showed that mice in the shCtrl groups had significantly better tumor growth delay and survival rates than mice in the shSNRPG groups. (D) Tumor weights were measured 30 days after injection (mean ± SD, *n* = 10, ^***^P < 0.001). (E) hematoxylin and eosin staining and immunohistochemistry analysis of proliferating cell nuclear antigen expression in tumors developed from U87MG/shSNRPG cells and from U87MG/shCtrl-transfected cells. Semiquantitative analysis of the stained sections was performed using light microscopy to calculate the immunoreactive score (mean ± SD, *n* = 3, ^**^P < 0.001).

To confirm whether *SNRPG* had any impact on tumorigenesis, shCtrl/shSNRPG-transfected U87MG cells were injected into nude mice, and all animals developed xenograft tumors at the inoculation site. **Figure 3A** and **3B** show that cancer development in the shSNRPG group was sluggish compared to that in the control group (upper panel), while the body weights of mice in the 2 groups were barely altered (lower panel) (^***^P < 0.001; ^****^P < 0.0001). Furthermore, *in vivo* fluorescence imaging was performed, showing that the shCtrl group mice had more extended cancer growth interruptions than the mice in the shSNRPG group (**Figure 3C**). Up to 30 days after injection, the typical tumor mass in the shSNRPG group was less than that in the shCtrl group (^***^P < 0.001) (**Figure 3D**). Overall, these results showed that functional SNRPG damage slowed down GBM tumorigenesis. The cancer cells in the shCtrl group were uneven and had ample cytoplasms, large and deformed nuclei, and high nucleocytoplasmic ratios (**Figure 3E**). In addition, nuclear pleomorphism and nucleoli were prominent. Cells with amphinucleoli and mitotic cells were also observed. In contrast, in the shSNRPG group, amphinucleoli and mitotic cells were rarely observed, and the size of the nucleolus was reduced. We have also demonstrated that the tumors arising from U87MG-shSNRPG cells exhibited inferior PCNA staining than tumors arising from shCtrl-transfected U87MG cells (^**^P < 0.01), as distinguished by immunohistochemistry (**Figure 3E**).

### SNRPG suppression sensitizes human GBM cells to temozolomide

**Figure 4:**
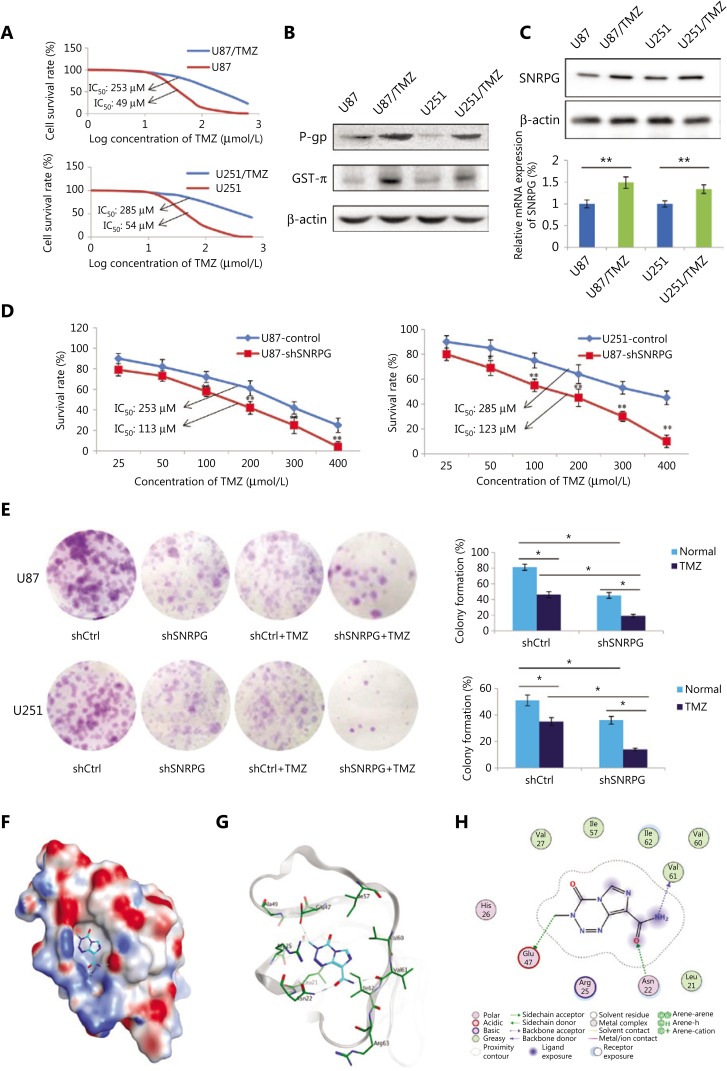
*SNRPG* suppression sensitizes human glioblastoma multiforme cells to temozolomide (TMZ). (A) The MTT assay was used to determine the viability of U87/TMZ and U251/TMZ cells (IC_50_ for U87, 49 μM *vs.* 253 μM; IC_50_ for U251, 54 μM *vs.* 285 μM). (B) Western blot analysis of the drug resistance-related genes P-gp and GST-π in U251/TMZ and U87/TMZ cells. (C) Western blot analysis (upper panel) and RT-qPCR analysis (lower panel) of *SNRPG* in U251/TMZ and U87/TMZ cells. (D) The MTT assay was used to determine the cell viability after *SNRPG* knockdown. Downregulation of *SNRPG* significantly reduced the IC_50_ compared with that of the control group (113 μM *vs.* 253 μM in U87/TMZ cells; 123 μM *vs.* 285 μM in U251/TMZ cells; ^*^P < 0.05; ^**^P < 0.01). (E) A colony formation assay was used to determine the cell growth after *SNRPG* knockdown and TMZ treatment. ^*^P < 0.05; ^**^P < 0.01. The colonies were counted after staining with 0.1% crystal violet (Original magnification, 1×). (F–H) The binding mode of TMZ bound to SNRPG by molecular docking. (F) The electrostatic map surface of the SNRPG protein. Positively charged areas are colored blue, and negatively charged areas are colored red. (G) The three-dimensional binding mode of TMZ bound to SNRPG. Gray ribbons: receptor backbone. Cyan sticks: ligand TMZ. Green sticks: binding pocket residues. Blue dashes: electrostatic interactions (e.g., hydrogen bonds). (H) The two-dimensional binding mode of TMZ bound to SNRPG.

TMZ-resistant U251 and U87 cells (U87/TMZ and U251/TMZ) were generated by treatment with increasing TMZ concentrations. The cells were incubated with various concentrations of TMZ to determine the IC_50_ value, and the IC_50_ of parental cells was shown to be lower than that of TMZ-resistant cells (**Figure 4A**). In addition, as presented in **Figure 4B**, the genes related to drug resistance, comprising P-gp and GST-π, were augmented in TMZ-resistant cells compared to those in the parental cells (**Figure 4B**). We also investigated the expression of *SNRPG* in drug-resistant GBM cells by Western blot and RT-qPCR, which showed that the expression of *SNRPG* was significantly increased in TMZ-resistant cells compared to that in parental cells (**Figure 4C**), demonstrating that downregulation of *SNRPG* might participate in GBM drug resistance.

We further investigated whether downregulating SNRPG expression repressed TMZ resistance in TMZ-resistant cells. The SNRPG deficiency suggested a decreased IC_50_ compared to that in the control group (^*^P < 0.05; ^**^P < 0.01) (**Figure 4D**). Furthermore, a colony assay was performed to assess the role of SNRPG in the proliferation of TMZ-resistant cells, which showed that SNRPG suppression suppressed cell proliferation (^*^P < 0.05; ^**^P < 0.01) (**Figure 4E**). In a similar manner, inhibition of SNRPG expression made the cells more sensitive to TMZ, as determined by reduced numbers of colonies compared to that of cells transfected with the scrambled control (**Figure 4E**).

To further confirm the direct connection between SNRPG and TMZ, docking simulations were conducted to predict the binding mode and binding affinity of the ligand TMZ with the SNRPG receptor. In the docking process, the top 20 scoring poses were retained for further analysis. Combining visual inspection and empirical analysis, we selected the most representative pose for the ligand TMZ, whose 3D and 2D binding modes are depicted in **Figure 4G** and **Figure 4H**. The electrostatic map of the protein surface was calculated and plotted in **Figure 4F**.

As indicated by the binding modes, the ligand TMZ bound strongly to the receptor, mainly through hydrogen bonds and hydrophobic interactions. It formed hydrogen bonds with the sidechains of Asn22 and Glu47 as well as with the backbone of Val61 (**Figure 4H**). In addition, hydrophobic interactions with the surrounding nonpolar residues, including Leu21, Val27, Ile57, Val60, and Ile62, were observed.

### SNRPG suppression may critically affect the cell cycle progression in GBM cells *via* Myc and p53 signaling

**Figure 5:**
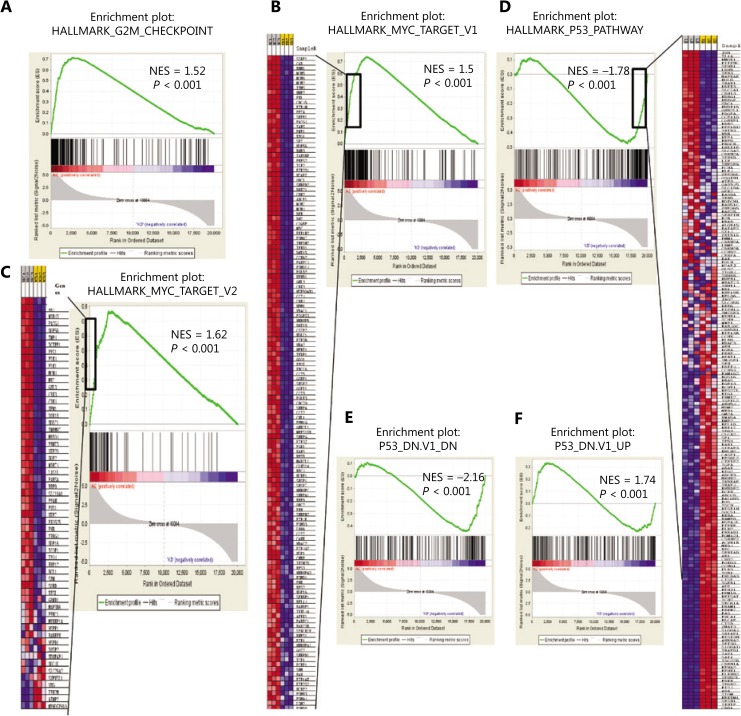
Bioinformatics analysis indicated that *SNRPG* inhibition affected Myc and downstream p53. (A) Gene set enrichment analysis (GSEA) was performed with GSEA tools to determine whether particular gene sets were significantly enriched in differentially expressed genes regulated by *SNRPG*. The normalized enrichment scores (NES) are shown in the plot. The genes affected by silencing *SNRPG* in glioblastoma multiforme cells were enriched in the G2/M checkpoint pathway signature. (B–F) GSEA plot of the Cancer Genome Atlas gene signatures in shCtrl or shSNRPG glioma cells. Gene expression profile data were obtained by cDNA microarray using glioma cells expressing shCtrl or shSNRPG. The NES is shown in the plot. Further GSEA revealed that several gene set classifications, as annotated by GSEA collections, were significantly enriched. Specifically, *SNRPG* inhibition could cause significant Myc inhibition (B–C) and p53 activation (D–F).

To identify whether any specific gene sets were possibly augmented in differentially expressed genes controlled by SNRPG, *GSEA* was performed using GSEA tools^35^. Notably, *SNRPG* suppression significantly enriched the G2/M checkpoint (**Figure 5A**), and numerous GSEA gene set categories, as determined by the GSEA collections, which were possibly augmented. Intriguingly, *SNRPG* suppression caused significant Myc inhibition (**Figure 5B and 5C**) and p53 activation (**Figure 5D–5F**), indicating that both Myc and p53 were downstream targets of SNRPG.

### SNRPG influences Myc cellular localization and Myc-dependent p53 activation

**Figure 6:**
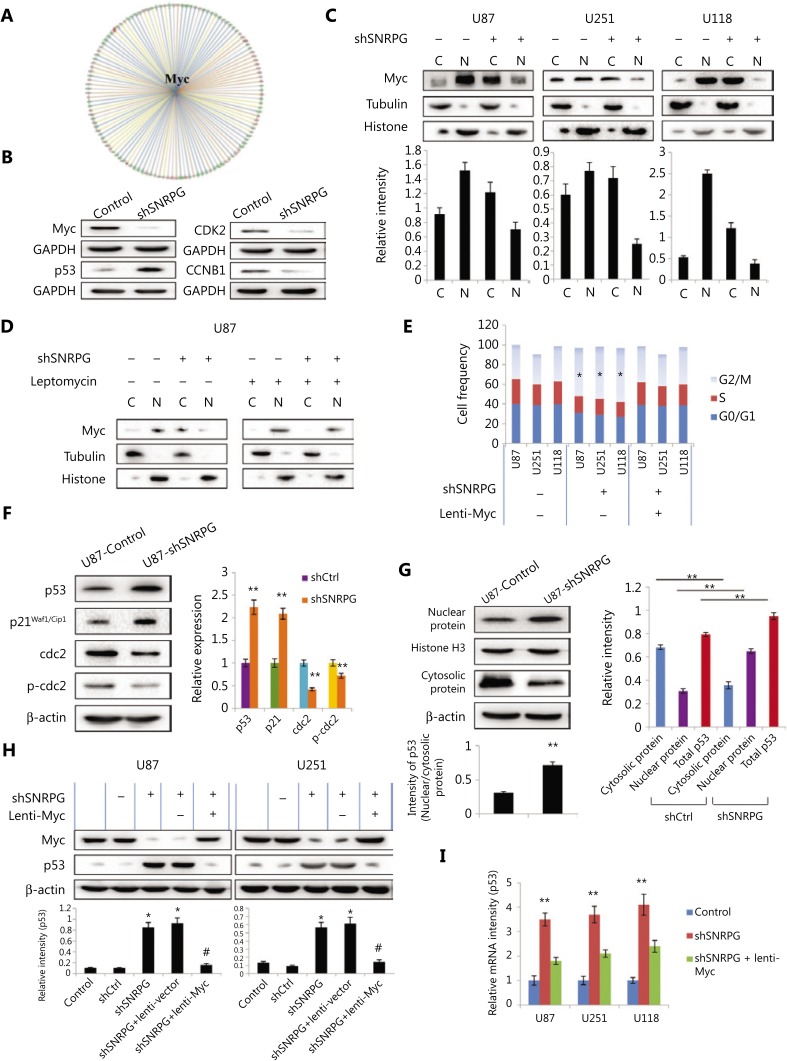
The results of analyzing microarray data regarding Myc function and downstream Myc-mediated cell cycle-associated proteins were further validated. (A) The knowledge-based interactome surrounding the regulation of Myc was examined using ingenuity pathway analysis (IPA) and overlaid with microarray data with a 1.5-fold change cut-off. (B) The expression levels of downstream cell cycle-associated proteins were further validated. First, Western blot confirmed that Myc and p53 were downregulated in *SNRPG*-silenced U87 cells compared with that in shCtrl-transduced cells. In addition, the knockdown of *SNRPG* in U87MG cells also decreased the expression of other cell cycle-associated proteins, such as CDK2 and CCNB1. (C) *SNRPG* controls the subcellular localization of Myc. The levels of Myc, histone H3, and tubulin in the cytoplasmic (Cy) and nuclear (Nu) fractions of cells expressing a scramble or *SNRPG*-targeted shRNA, as assessed by Western blot, are shown, *n* = 3. (D) Western blot analysis of Myc, tubulin, and histone in the nuclear and cytoplasmic fractions of control and shSNRPG-expressing U87MG cells incubated with 0 or 5 ng of leptomycin B (LMB, 8 h). (E) *SNRPG* suppression increases cell cycle arrest via a Myc-dependent pathway. The cell cycle distribution was confirmed in *SNRPG* knockdown cells and in lentivirus-infected U87MG cells overexpressing Myc. ^*^P < 0.05, *n* = 3. (F–I) The levels of *SNRPG* correlated inversely with p53 expression *in vitro* and *in vivo*, which affected the sensitivity of GBM cells to TMZ. (F) Western blot analysis showed that *p53* and its target genes, especially *p21*, were upregulated in *SNRPG*-silenced U87MG cells compared with those in shCtrl-transduced cells (mean ± SD, *n* = 3, ^**^P < 0.001 *vs.* Ctrl or shCtrl). (G, left panel) Representative Western blot analyses of p53 nuclear/cytoplasmic expression in control and shSNRPG-transfected U87MG cells *in vitro*. Bar = 50 μm, *n* = 3. (G, right panel) Average subcellular distribution of p53 (nuclear and cytosolic protein expression) and total p53 expression in U87MG cells from U87 tumor cells grown as xenografts in the shCtrl and shSNRPG groups. ^**^P < 0.01, *n* = 3. (H–I) *SNRPG* suppression increased the p53 levels via a Myc-dependent pathway. The protein levels (H) and mRNA levels (I) of p53 were confirmed in *SNRPG* knockdown cells and in lentivirus-infected U87MG cells overexpressing Myc. ^*^P < 0.05 and ^**^P < 0.01 *vs.* the control group; ^#^*P* < 0.05 *vs.* the shSNRPG group, *n* = 3.

Bioinformatic analysis further revealed downstream molecular targets regulated by Myc (**Figure 6A**). The knowledge-dependent interactome associated with Myc regulation was analyzed by ingenuity pathway analysis (IPA) and overlaid with microarray data with a 1.5-fold change cut-off. In addition, downstream targets of Myc and p53 were assessed via bioinformatics analysis (**Supplementary Table S2**). To confirm the outcomes of microarray and IPA analyses, Western blot was performed to evaluate the expression of a group of representative proteins. The outcomes suggested that knockdown of *SNRPG* in U87MG cells diminished Myc expression (**Figure 6B**), confirming the microarray data, which further verified the regulatory effect of SNRPG on Myc. In addition, changes in the expression levels of various other cell cycle-associated proteins were confirmed (**Figure 6B**). Specifically, p53, CKD2, and CCNB1 expression was found to be significantly changed in *SNRPG*-silenced U87MG cells. These experimental and bioinformatics analyses showed that SNRPG regulated the cell cycle control of chromosomal replication, cyclins, and the cell cycle regulation pathway in human U87MG glioblastoma cells, and suggested that the effects of *SNRPG* downregulation on the cell cycle as well as on apoptosis were most likely dependent on the comprehensive regulation of cellular functions.

Myc is a transcription factor that binds to DNA at E-Box sequences to initiate the transcription of numerous target genes^36^. We next examined the effect of *SNRPG* knockdown on Myc localization. Myc was observed predominantly in the nucleus (**Figure 6C**). In *SNRPG*-knockdown cells, however, Myc was mainly present in the cytoplasm, while its expression was decreased in the nucleus (**Figure 6C**). Incubation of cells with leptomycin, a pharmacological blocker of nuclear export^37^, had no effect on the nuclear localization of Myc in control cells, but blocked the cytoplasmic accumulation of Myc in *SNRPG*-knockdown cells (**Figure 6D**). These results showed that loss of SNRPG expression increased the export of Myc to the cytoplasm. In addition, we also investigated whether the effects of SNRPG loss on cell cycle progression were Myc-dependent. The results indicated that the cell cycle arrest caused by *SNRPG* knockdown was reversed in lentivirus-infected U87MG cells overexpressing Myc (^*^P < 0.05) (**Figure 6E**). These results showed that loss of *SNRPG* led to decreased levels of Myc, which might significantly contribute to the lack of cell cycle progression observed in *SNRPG*-knockdown cells.

RT-qPCR together with Western blot further confirmed that the expression levels of p53 and its target genes, especially p21, were upregulated in *SNRPG*-silenced U87MG cells (**Figure 6F**). In addition, silencing *SNRPG* repressed the phosphorylation and expression of cdc2, which may have led to its effect on prompting G2/M phase arrest (^**^P < 0.01) (**Figure 6F**). In addition, to more fully addressing the relationships between *SNRPG* and p53 localization and expression, both control and SNRPG-suppressed U87MG cells grown *in vitro* were examined by Western blot. The p53 was primarily cytosolic in control U87MG cells grown in culture, with a relative lack of p53 staining. In contrast, in *SNRPG*-knockdown cells, p53 exhibited nuclear localization in association with increased expression (^**^P < 0.01) (**Figure 6G, left panel**). The same results were observed *in vivo*, as U87MG tumor cells grown as xenografts expressed cytosolic p53, but little nuclear p53, whereas tumors derived from U87MG cells in which *SNRPG* was knocked down exhibited increased expression of p53 in the nucleus (^**^P < 0.01) (**Figure 6G**, right panel).

The transcriptional activity of Myc involves its binding to DNA at E-Box sequences to initiate the transcription of numerous target genes, a process that might involve p53^36^. We also investigated whether the effects of SNRPG loss on p53 levels were Myc-dependent, which showed that the increased p53 levels caused by *SNRPG* knockdown were reversed in lentivirus-infected U87MG cells overexpressing Myc (**Figure 6H**). In addition, overexpression of Myc in *SNRPG*-knockdown cells decreased the expression of p53 (**Figure 6I**). These results showed that the loss of SNRPG led to decreased levels of Myc. Subsequent stimulation of p53 and increased p53 protein levels could significantly contribute to the lack of cell cycle progression in *SNRPG*-knockdown cells.

### SNRPG suppression sensitizes GBM cells to TMZ through Myc and p53 signaling

**Figure 7:**
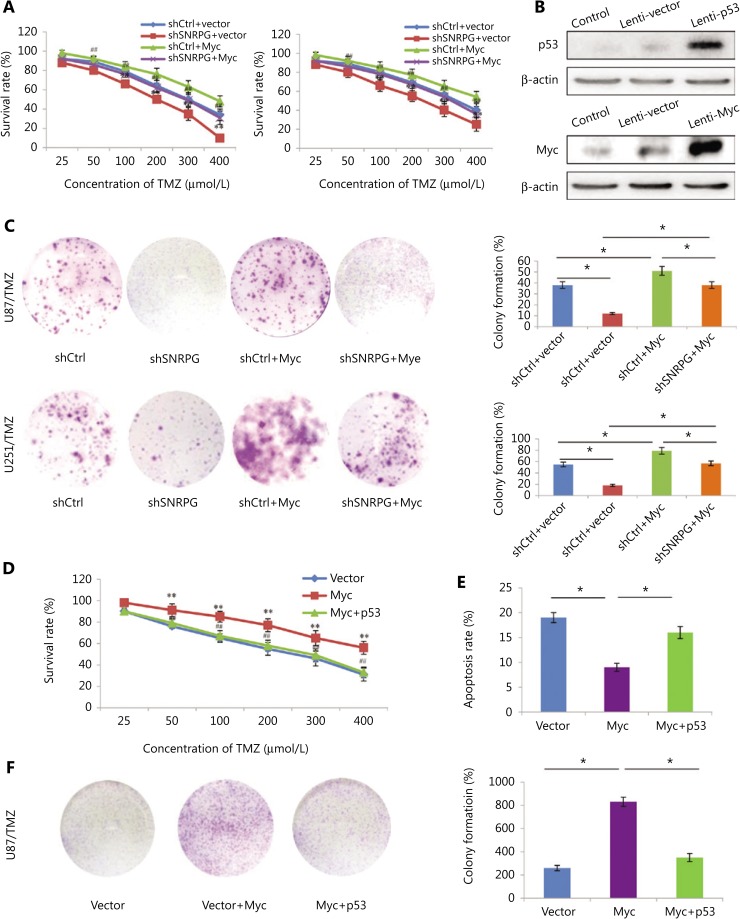
Overexpression of Myc reverses the inhibitory effects of SNRPG downregulation, and activation of p53 signaling reverses the oncogenic effects of Myc in temozolomide-resistant glioblastoma multiforme cells. (A) The MTT assay was used to determine the cell viability after knocking down SNRPG and overexpressing Myc. The IC_50_ was determined in U87 (shCtrl + vector group: 297 μM; shSNRPG + vector group: 201 μM; shCtrl + Myc group: 372 μM; shSNRPG + Myc group: 289 μM), and U251 cells (shCtrl + vector group: 321 μM; shSNRPG + vector group: 226 μM; shCtrl + Myc group: 409 μM; shSNRPG + Myc group: 313 μM). ^*^P < 0.05; ^**^P < 0.01 *vs.* the shCtrl+vector group; ^##^*P* < 0.01 *vs.* the shSNRPG + vector group. (B) The Myc and p53 protein abundances in GBM cells, as indicated, were determined by immunoblot analysis. β-actin was used as the loading control. (C) A colony formation assay performed in the absence of TMZ was used to determine the cell growth after knocking down SNRPG and overexpressing Myc. The colonies were counted after staining with 0.1% crystal violet (Original magnification, 1×). (D) The MTT assay was used to determine the cell viability after Myc transfection and p53 overexpression (IC_50_ for Vector, 253 μM; IC_50_ for Myc, 497 μM; IC_50_ for Myc + p53, 295 μM). ^**^P < 0.01 *vs.* the vector group; ^##^*P* < 0.01 *vs.* the Myc group. (E) An apoptosis assay was used to assess cell apoptosis after Myc transfection and p53 overexpression. ^*^P < 0.05. (F) A colony formation assay was used to determine cell growth after Myc transfection and p53 overexpression. ^*^P < 0.05. The colonies were counted after staining with 0.1% crystal violet (Original magnification, 1×).

**Figure 8:**
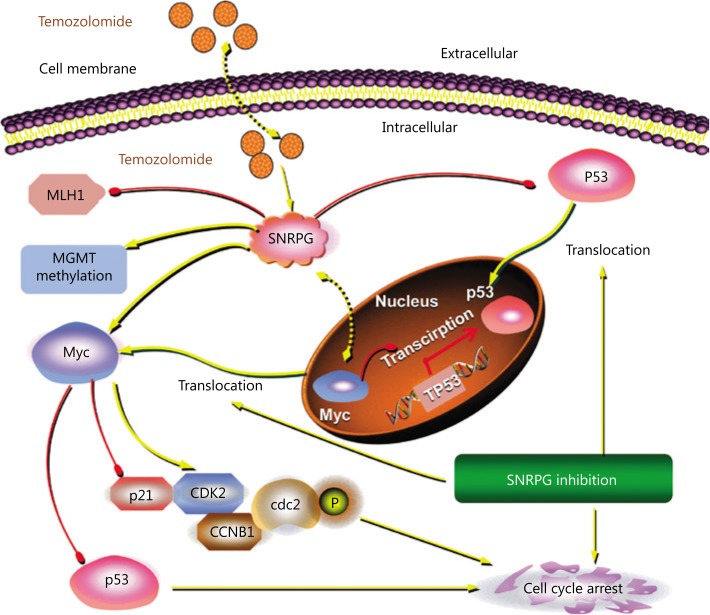
Schematic diagram of the molecular mechanisms of SNRPG-mediated p53 targeting of TMZ-induced glioblastoma multiforme (GBM) growth inhibition. The SNRPG-mediated inhibitory effect on glioma cells might have been due to the direct suppression of Myc as well as p53 and the ensuing modification of their downstream molecules including p21, CDK2, CCNB1, and cdc2, possibly promoting apoptosis and cell cycle arrest by interacting directly or indirectly with related proteins. Specifically, SNRPG suppression promoted the cytosolic translocalization of Myc, as well as the nuclear translocalization of p53. Furthermore, knockdown of SNRPG in GBM cells promoted the inhibitory effect of TMZ on cell proliferation by targeting Myc through the p53 signaling pathway, suggesting its regulatory effect on TMZ sensitivity. Besides, SNRPG decreased the protein expression of the mismatch repair (MMR) protein MLH1 and increased MGMT methylation, which could all be well-established mechanisms of TMZ resistance. These findings could indicate that SNRPG-driven systemic Myc-regulated p53 activation and sensitization of GBM cells to TMZ are highly promising strategies for treating glioma.

We previously showed that Myc is a target of SNRPG and that downregulating SNRPG decreases the expression of Myc. Furthermore, we evaluated whether SNRPG deficiency sensitized GBM cells to TMZ through Myc. Overexpression of Myc significantly augmented the endurance rate of TMZ-resistant cells after TMZ treatment, whereas SNRPG suppression significantly inhibited the survival of TMZ-resistant cells following TMZ treatment (**Figure 7A**). However, the inhibition induced by SNRPG suppression was abolished by Myc overexpression (^*^P < 0.05; ^**^P < 0.01 *vs.* the shCtrl + vector group; ^#^*P* < 0.05; ^##^*P* < 0.01 *vs.* the shSNRPG + vector group) (**Figure 7A**). The evidence for Myc and p53 overexpression is shown in **Figure 7B**. Comparable outcomes were also obtained in the colony formation assay (**Figure 7C**). In addition, we overexpressed p53 in U87/TMZ cells, and as shown in **Figure 7C**, activation of p53 signaling reversed the oncogenic effects of Myc as determined by the cell viability (^**^P < 0.01 *vs.* the vector group; ^##^*P* < 0.01 *vs.* the Myc group) (**Figure 7D**), apoptosis (^*^P < 0.05) (**Figure 7E**), and colony formation ability (^*^P < 0.05) (**Figure 7F**) of U87/TMZ cells. These results indicated that SNRPG suppression sensitized GBM cells to TMZ by targeting Myc via the p53 signaling cascade (**Figure 8**).

## Discussion

Current data suggest that *SNRPG* expression in glioma tissues is higher than that in healthy control tissues and that suppressing *SNRPG* expression reduces the proliferation of GBM by inhibiting cell proliferation and promoting apoptosis and G2/M cell cycle arrest. The *SNRPG*-mediated inhibitory effect on glioma cells might have been due to the direct suppression of Myc as well as p53 and the ensuing modification of their downstream molecules, possibly promoting apoptosis and cell cycle arrest by interacting directly or indirectly with related proteins. Furthermore, knockdown of *SNRPG* in U87MG cells promoted the inhibitory effect of TMZ on cell proliferation, suggesting its regulatory effect on TMZ sensitivity; current studies indicate that *SNRPG* suppression sensitized GBM cells to TMZ by targeting Myc through the p53 signaling pathway. These findings suggest the high potential of *SNRPG* as a chemotherapeutic target for treating malignant glioma.

Myc is overexpressed in numerous tumors and plays leading roles in both tumor cell cycle regulation and pathogenesis^4,38,39^. Myc promotes tumor cell production by regulating the cell cycle^4^, and Myc suppression is a favorable approach for treating both lung cancer^40,41^ and insulinoma^42^ by promoting intense tumor deterioration. Only a few slight side effects in normal tissues have been documented, and the complications of resistance with other targeted treatments are avoided. Although the direct pharmacological suppression of Myc is not currently an option, the function of Myc can potentially be directly prevented, as recent studies have reported the use of peptides derived from Myc itself^43^. Myc function can be indirectly prevented, as established by bromodomain blockers^38^. Current data suggest that the loss of *SNRPG* could lead to decreased levels of Myc, which might significantly contribute to cell cycle progression deficits, and this phenomenon could be induced by increasing the export of Myc to the cytoplasm. However, how *SNRPG* regulates Myc localization requires further investigation, and further studies should thus focus on elucidating the exact mechanisms controlling the effects of SNRPG on Myc, and whether the effects of Myc suppression are documented in different types of tumors remains to be recognized.

Intriguingly, the p53 protein halts the cell cycle at numerous points under certain conditions, such as beyond the DNA damage restoration point and upon activation of apoptotic cascades^44^. The stimulation of p53 can transcriptionally augment the expression levels of its target genes, especially p21^45^. In the current study, silencing *SNRPG* augmented G2/M phase arrest and amplified expression of the p53 and p21^Waf1/Cip1^ proteins. In addition, cdc2 activity is reportedly vital for progression into mitosis and into the S phase of the cell cycle^46^. We have also reported that silencing *SNRPG* represses the phosphorylation and expression of cdc2, which may lead to its promotion of G2/M phase arrest. These results are consistent with previous results. Furthermore, current data indicated that silencing *p53* reversed the decreased expression induced by *SNRPG* knockdown. Although p53 may exert an important effect on regulating the mesenchymal (MES) identity of GBM cells mediated by *SNRPG*, other probable processes participating in the effect of silencing *SNRPG* on cell production in p53 mutant or p53 null cells must exist, and this topic needs further investigation.

Importantly, resistance to chemotherapy remains an enormous clinical problem^47^. The current study also indicated that *SNRPG* expression was increased in TMZ-resistant GBM cells, and downregulating *SNRPG* made the cells resistant to TMZ. Overall, the current study confirmed that *SNRPG* suppression sensitized GBM cells to TMZ by targeting Myc via the p53 signaling cascade (**Figure 8**). Our findings showed that suppression of *SNRPG* in resistant GBM cells might be a valuable method for overcoming essential drug resistance.

In this study, various problems potentially need to be addressed. First, the present study established that *SNRPG* expedited cell cycle development and thus stimulated cell production. Moreover, this function was the outcome of the regulation of various signaling cascades, as suggested by microarray data. Although we have not studied the functions of other genes, other genes with altered expression in *SNRPG*-silenced cells are potentially important in GBM, and further investigation is warranted. Second, the xenograft experimental results demonstrated that reducing *SNRPG* expression decelerated tumor development in an animal model, but the effects of silencing *SNRPG* on mouse mortality were not assessed and should be further addressed in future studies. Finally, *SNRPG* is known to play important roles in the processing of the 3' ends of histone transcripts or histone modifications (http://www.genecards.org/) and potentially in protein translation during replication. As histone modifications can impact alternative splicing, we believe that an *SNRPG*-driven histone-based system might help elucidate details regarding the collection of alternative splicing arrays in cells and tissues^48–50^, which may be the direction of our future studies.

## Conclusions

Although substantial efforts have been made in exploiting new treatment agents, novel treatments for glioma, an illness with a particularly negative prognosis, are urgently needed, as this disease remains one of the most fatal malignancies. Assessment of the *SNRPG*-derived oncogenic effect might contribute to the discovery of new targets for the treatment of malignant glioma. Our work was designed to elucidate the anticancer mechanisms of *SNRPG* suppression in malignant glioma. In conclusion, our data indicated that *SNRPG*-driven systemic Myc-regulated p53 activation and sensitization of GBM cells to TMZ are highly promising strategies for treating glioma. Additionally, recognition of *SNRPG*-specific inhibitors may create a novel model for the detection and advancement of molecularly targeted treatments for cancers, including GBM. These conclusions provide robust evidence for the potential of *SNRPG* as a new candidate for malignant glioma treatment.
