# Supplementary material for: Downregulation of SNRPG induces cell cycle arrest and sensitizes human glioblastoma cells to temozolomide by targeting Myc through a p53-dependent signaling pathway
Source: Cancer Biol Med. 2020 Feb 15;17(1):112–31. doi: 10.20892/j.issn.2095-3941.2019.0164 (PMC7142844; doi:10.20892/j.issn.2095-3941.2019.0164)
Supplement: Supplementary file 1 [file cbm-17-112-s001.pdf]

Supplementary materials

Table S1 General feature of patients

Sample No.	Age	Gender	Family history of cancer (Y/N)	History of smoking (Y/N)	History of drinking (Y/N)	Diagnosis	Tumor size ≥ 4.5cm (Y/N)	Tumor location (supratentorial/ subtentorial)
G-0004	33	Female	No	No	No	GBM (WHO IV)	No	Supratentorial
G-0115	60	Male	No	No	No	GBM (WHO IV)	No	Supratentorial
G-0132	48	Male	No	Yes	Yes	GBM (WHO III-IV)	Yes	Supratentorial
G-0266	41	Female	No	No	No	GBM (WHO IV)	Yes	Supratentorial
G-0285	62	Female	No	No	No	GBM (WHO IV)	No	Supratentorial
G-0396	55	Female	No	No	No	GBM (WHO IV)	Yes	Supratentorial
G-0558	55	Female	No	No	No	GBM (WHO IV)	Yes	Supratentorial
G-0666	65	Male	No	No	No	GBM (WHO IV)	Yes	Supratentorial
G-0729	36	Male	No	Yes	No	GBM (WHO IV)	Yes	Supratentorial
G-0821	54	Male	No	No	No	GBM (WHO IV)	Yes	Supratentorial
G-0839	61	Male	No	No	No	GBM (WHO IV)	Yes	Supratentorial
G-0940	57	Female	No	No	No	GBM (WHO IV)	No	Supratentorial
G-0944	60	Female	No	No	No	GBM (WHO IV)	Yes	Supratentorial
G-0996	67	Male	No	No	No	GBM (WHO IV)	Yes	Supratentorial

Table S2 Downstream targets of Myc and p53 assessed via bioinformatics analysis

Downstream targets of Myc assessed *via* bioinformatic analysis after knocking down SNRPG			
Target molecules in dataset	Regulation	Fold_change	*P*
YAP1	Down	-2.2004654	0.000899037
WLS	Up	2.726611	6.40171E-07
WISP1	Up	3.8561678	6.47337E-06
VEGFA	Down	-2.4606857	5.41824E-07
VCAM1	Up	31.123684	2.31657E-06
VARS	Down	-2.152252	0.00037761
UBE2S	Down	-2.0267875	1.7197E-05
UBE2C	Down	-3.1527188	1.35925E-06
TYMS	Down	-2.8719566	2.08657E-06
TXNIP	Up	2.5055113	2.4035E-06
TP53I3	Up	3.2173285	3.44497E-05
TMEM97	Down	-2.6563091	0.00019609
STMN1	Down	-2.428075	3.16331E-05
SPARC	Up	2.983737	5.69005E-06
SOD2	Up	2.3653908	5.75501E-08
SMAD1	Up	2.5486372	8.24186E-06
SLC38A1	Down	-2.1260705	0.000338194
SGK1	Up	2.060982	1.9894E-05
SERPINH1	Up	2.0949132	3.29312E-06
SERPINE1	Down	-2.776294	1.63496E-06
SERPINA1	Up	3.655164	3.9032E-07
SAT1	Up	2.3773856	1.38163E-06
RRM2	Down	-2.6255248	7.52328E-07
RCC1	Down	-2.737947	1.65928E-07
RBP1	Up	2.9123752	6.93317E-05
RARRES1	Up	4.272019	0.000169545
RAD51	Down	-2.2705245	9.49069E-05
RAB40B	Up	3.1059167	2.69713E-06

Continued

Downstream targets of Myc assessed *via* bioinformatic analysis after knocking down SNRPG			
Target molecules in dataset	Regulation	Fold_change	*P*
PSAT1	Down	-2.7941062	4.30426E-05
PRDM1	Up	2.4411225	3.35728E-05
PLK1	Down	-2.3198814	0.000164844
PFAS	Down	-2.2056475	1.46729E-06
PDGFRA	Up	3.8465552	0.000319564
PCDH18	Up	4.690889	4.71284E-05
NRP1	Up	2.192101	3.39564E-05
NDRG1	Down	-2.0377207	5.49884E-05
MYC	Down	-2.3531933	6.84799E-06
MTFR2	Down	-3.1721334	7.32363E-06
MRE11A	Down	-2.422145	9.68115E-06
MKI67	Down	-3.6224332	2.8842E-06
MIR17HG	Down	-2.792616	0.000221759
MGP	Up	22.994284	3.3942E-07
MFAP1	Down	-2.154815	1.66954E-05
MCM7	Down	-3.4240937	1.39583E-06
MCM6	Down	-2.7770162	1.12485E-06
MCM5	Down	-2.7076702	0.000152585
MAN2A1	Up	2.584694	5.13991E-06
MAD2L1	Down	-3.0069954	3.91006E-05
LXN	Up	2.1453402	2.22587E-06
LGMN	Up	2.3995295	1.73229E-05
KRT7	Up	2.1850588	0.000203507
ITM2B	Up	2.4764104	1.41161E-08
ID3	Up	4.5758276	2.72157E-06
ICAM1	Up	2.7644155	1.96913E-05
HMGA1	Down	-4.1570177	6.52401E-07
HES1	Up	4.7940874	8.81984E-05

Continued

Table S2 Continued

Downstream targets of Myc assessed *via* bioinformatic analysis after knocking down SNRPG			
Target molecules in dataset	Regulation	Fold_change	*P*
HAS2	Down	-2.077807	0.000111374
GLG1	Up	2.0773172	4.01057E-06
GFPT1	Down	-4.121707	3.02735E-05
GCSH	Down	-2.9161048	1.13144E-05
GCLM	Down	-2.076191	6.14521E-07
GBP2	Up	2.9921346	0.000191824
GART	Down	-2.694237	0.000257483
FOXM1	Down	-3.0084047	2.38538E-05
FOSL1	Down	-3.0467093	2.21821E-05
FOS	Up	2.3506725	1.93163E-05
FN1	Up	3.2369347	1.12496E-05
FGF5	Down	-2.0261834	0.000121213
FCGRT	Up	2.0499787	2.14653E-05
FAS	Up	2.1131759	2.28379E-05
EZH2	Down	-2.6572704	3.36057E-05
EXOSC8	Down	-2.339027	6.42808E-07
EMP1	Down	-2.6436226	5.68407E-05
EIF4EBP1	Down	-2.1822093	4.25906E-05
EIF3G	Up	2.0444875	0.000525781
EIF2S1	Down	-2.4059823	2.03661E-06
EGR1	Up	2.6178222	7.71939E-06
EFEMP1	Up	3.8927963	3.04738E-05
EBI3	Up	5.3947735	0.00029493
E2F1	Down	-2.6413052	0.000395762
DUSP6	Down	-2.6694174	1.71495E-05
DKK1	Down	-2.4766245	0.000258911
DHFR	Down	-2.5963402	2.21914E-05
CXCL10	Up	6.316949	8.62745E-06

Continued

Downstream targets of Myc assessed *via* bioinformatic analysis after knocking down SNRPG			
Target molecules in dataset	Regulation	Fold_change	*P*
CTBS	Down	-2.0393775	0.000428111
CSRP2	Down	-2.0059276	2.51977E-06
CRYAB	Up	7.0759397	3.75693E-05
CPD	Up	2.2487748	1.51939E-06
COL5A2	Up	5.0252233	1.22958E-06
COL5A1	Up	2.1802685	7.51787E-06
COL3A1	Up	6.0942416	2.7127E-05
COL1A2	Up	3.3868077	2.98091E-07
COL14A1	Up	3.668856	0.00015045
CLEC3B	Up	3.7030663	1.25343E-06
CKS2	Down	-2.22107	2.06846E-07
CHEK1	Down	-2.2617967	6.8628E-05
CEBPD	Up	8.443227	6.37108E-06
CDKN1A	Up	2.0020273	3.1422E-05
CDK6	Up	2.2414362	2.83847E-05
CDK2	Down	-2.3414066	0.000723858
CDK1	Down	-3.3894331	3.41338E-08
CDCA7	Down	-2.5425134	3.25259E-05
CDC25C	Down	-2.9542015	1.06227E-05
CDC25A	Down	-2.0147529	0.002159371
CDC20	Down	-3.6122062	1.22727E-07
CD274	Down	-2.3226845	0.002961726
CCNB2	Down	-2.8234894	1.19585E-06
CCNB1	Down	-3.2859113	2.33511E-07
CCNA2	Down	-3.3991127	1.46568E-06
C9orf3	Up	2.142429	0.000119559
BUB1B	Down	-3.9987106	4.8566E-06
BUB1	Down	-3.664866	1.47275E-06

Continued

Table S2 Continued

Downstream targets of Myc assessed *via* bioinformatic analysis after knocking down SNRPG			
Target molecules in dataset	Regulation	Fold_change	*P*
BRCA1	Down	-3.013453	2.99571E-05
BIRC5	Down	-4.0315733	1.76944E-06
BDNF	Up	2.0975358	3.10914E-06
BCL6	Up	2.1456041	0.000507154
BCAT1	Down	-2.603652	1.53501E-06
BBC3	Up	2.1616032	0.000223194
AURKB	Down	-2.6026242	7.70047E-06
ASNS	Down	-2.0371935	4.61309E-06
ARL6IP1	Down	-2.4931567	2.49777E-07
ARHGAP22	Down	-2.2010329	1.57562E-06
ANGPT1	Up	3.9937463	0.000218086
AKAP12	Down	-2.8823323	1.32047E-06
ADAMTS1	Down	-2.3186843	3.74103E-07
ABCE1	Down	-2.2096348	4.0258E-05

Downstream targets of p53 assessed *via* bioinformatic analysis after knocking down SNRPG			
Target molecules in dataset	Regulation	Fold_change	*P*
ACP2	Up	2.5898085	3.46048E-06
ACSL3	Down	-2.747913	6.88464E-06
ACTA2	Up	2.0995708	6.24381E-06
ADCK3	Up	2.347324	0.003031234
AKAP12	Down	-2.8823323	1.32047E-06
ALDH4A1	Up	2.4930303	0.000318248
ANGPT1	Up	3.9937463	0.000218086
ANLN	Down	-4.2728977	1.81942E-05
ARL6IP1	Down	-2.4931567	2.49777E-07
ASF1B	Down	-2.924686	3.86935E-06

Continued

Downstream targets of p53 assessed *via* bioinformatic analysis after knocking down SNRPG			
Target molecules in dataset	Regulation	Fold_change	*P*
ASNS	Down	-2.0371935	4.61309E-06
ASPM	Down	-4.345241	2.71048E-06
ATAD2	Down	-3.2450206	2.73272E-05
AURKA	Down	-3.0987566	6.72217E-07
AURKB	Down	-2.6026242	7.70047E-06
AXIN2	Up	2.3022826	0.000557813
BAK1	Up	2.0840688	0.000638436
BBC3	Up	2.1616032	0.000223194
BCL2L11	Up	5.340361	1.4619E-05
BDKRB2	Up	2.5592594	4.44557E-05
BID	Up	2.4654102	9.44967E-07
BIRC5	Down	-4.0315733	1.76944E-06
BMP1	Up	3.4533818	2.53152E-05
BRCA1	Down	-3.013453	2.99571E-05
BRCA2	Down	-2.852761	2.28877E-05
BTG2	Up	2.1304529	3.59177E-05
BUB1	Down	-3.664866	1.47275E-06
BUB1B	Down	-3.9987106	4.8566E-06
CA9	Down	-2.1404824	2.70905E-06
CCL2	Up	6.9680066	2.9082E-07
CCNA2	Down	-3.3991127	1.46568E-06
CCNB1	Down	-3.2859113	2.33511E-07
CCNB2	Down	-2.8234894	1.19585E-06
CDC20	Down	-3.6122062	1.22727E-07
CDC25A	Down	-2.0147529	0.002159371
CDC25C	Down	-2.9542015	1.06227E-05
CDC6	Down	-3.687949	9.97116E-07
CDC7	Down	-2.1907902	0.000464492

Continued

Table S2 Continued

Downstream targets of p53 assessed *via* bioinformatic analysis after knocking down SNRPG			
Target molecules in dataset	Regulation	Fold_change	*P*
CDK1	Down	-3.3894331	3.41338E-08
CDK2	Down	-2.3414066	0.000723858
CDKN1A	Up	2.0020273	3.1422E-05
CDKN3	Down	-4.096533	1.0441E-06
CDT1	Down	-2.8250837	8.81031E-05
CENPF	Down	-2.5941923	7.81122E-05
CEP55	Down	-4.4314423	1.77357E-05
CHEK1	Down	-2.2617967	6.8628E-05
CKAP2	Down	-2.1922245	1.45445E-05
CKB	Up	2.0246646	0.00010062
CKS1B	Down	-2.887445	4.66962E-05
CNN2	Down	-2.6031473	1.1283E-05
COL14A1	Up	3.668856	0.00015045
COL1A2	Up	3.3868077	2.98091E-07
COL3A1	Up	6.0942416	2.7127E-05
COL5A2	Up	5.0252233	1.22958E-06
CRYAB	Up	7.0759397	3.75693E-05
CTSF	Up	2.1767623	0.000418674
CXCL1	Up	2.8209019	7.21376E-06
DBF4	Down	-3.6854699	2.00798E-05
DDIAS	Down	-2.9275298	0.002405864
DDIT4L	Up	5.2929754	9.59775E-07
DDR1	Up	3.3824682	1.23232E-06
DHFR	Down	-2.5963402	2.21914E-05
DICER1	Up	2.3602622	2.18034E-05
DKK1	Down	-2.4766245	0.000258911
DLGAP5	Down	-3.9265223	1.49922E-05
DSN1	Down	-2.786994	1.21996E-05

Continued

Downstream targets of p53 assessed *via* bioinformatic analysis after knocking down SNRPG			
Target molecules in dataset	Regulation	Fold_change	*P*
DUSP6	Down	-2.6694174	1.71495E-05
DUT	Down	-2.1344292	0.000372482
E2F1	Down	-2.6413052	0.000395762
E2F8	Down	-2.1755638	1.73999E-05
EGF	Up	2.2057219	0.00019899
EGR1	Up	2.6178222	7.71939E-06
ENG	Up	2.5714345	0.000294865
ENPP2	Up	3.4637127	2.19034E-06
EPHX1	Up	2.0975645	6.01894E-05
ESPL1	Down	-2.6732528	1.67595E-06
EXO1	Down	-2.5579734	0.002804422
EZH2	Down	-2.6572704	3.36057E-05
EZR	Down	-2.5168595	4.72436E-05
F11R	Up	2.318464	2.48687E-05
FAM83D	Down	-3.2122777	2.72169E-05
FANCI	Down	-3.0667968	1.31616E-05
FAS	Up	2.1131759	2.28379E-05
FBXW7	Up	2.2539053	8.28855E-06
FEN1	Down	-2.4864187	1.55089E-05
FERMT2	Down	-2.0035534	5.38065E-06
FN1	Up	3.2369347	1.12496E-05
FOS	Up	2.3506725	1.93163E-05
FOSL1	Down	-3.0467093	2.21821E-05
FOXM1	Down	-3.0084047	2.38538E-05
FUCA1	Up	4.7821565	1.45953E-06
GART	Down	-2.694237	0.000257483
GLIPR1	Down	-2.279712	1.72457E-06
GMNN	Down	-2.4207106	1.38595E-06

Continued

Table S2 Continued

Downstream targets of p53 assessed *via* bioinformatic analysis after knocking down SNRPG			
Target molecules in dataset	Regulation	Fold_change	*P*
GNAI1	Up	2.3712525	1.11948E-05
GTSE1	Down	-3.179631	0.000123705
HAS2	Down	-2.077807	0.000111374
HJURP	Down	-2.9257886	4.46607E-06
HMGB2	Down	-2.4225783	1.96645E-06
HMGCS1	Down	-2.1236782	1.71473E-06
HMMR	Down	-4.2627034	3.08806E-06
ICAM1	Up	2.7644155	1.96913E-05
ID3	Up	4.5758276	2.72157E-06
IGDCC4	Up	3.4510975	2.31546E-06
IGFBP3	Up	4.6044054	4.75433E-07
IGFBP4	Up	2.587116	0.000130696
IGFBP5	Up	14.235849	9.1868E-06
IL1B	Down	-3.6559737	2.24477E-06
IL6	Up	2.2604623	5.30168E-07
ITGA2	Down	-2.00359	3.0551E-06
KIAA0101	Down	-3.5872898	7.89813E-05
KIF23	Down	-3.642677	0.000104343
KIFC1	Down	-3.3392606	2.3573E-05
KIT	Up	2.1119153	0.000167271
KNTC1	Down	-2.299394	2.53516E-06
KRT15	Down	-3.0376554	2.42634E-05
LAMA5	Up	2.2744126	2.76063E-05
LBR	Down	-2.9253728	1.75678E-06
LPIN1	Down	-3.7294765	6.3445E-07
MAD2L1	Down	-3.0069954	3.91006E-05
MAFB	Up	3.5815496	1.60634E-07
MAN2A1	Up	2.584694	5.13991E-06

Continued

Downstream targets of p53 assessed *via* bioinformatic analysis after knocking down SNRPG			
Target molecules in dataset	Regulation	Fold_change	*P*
MAP3K8	Up	3.197822	7.80446E-05
MCM2	Down	-3.3188963	4.27989E-07
MCM3	Down	-2.6946843	1.45363E-06
MCM4	Down	-2.8259888	2.29587E-05
MCM5	Down	-2.7076702	0.000152585
MCM6	Down	-2.7770162	1.12485E-06
MCM7	Down	-3.4240937	1.39583E-06
MELK	Down	-3.3216226	6.23091E-07
MGST2	Up	3.2562354	1.54722E-06
MIR17HG	Down	-2.792616	0.000221759
MIS18BP1	Down	-2.1686413	3.06466E-05
MKI67	Down	-3.6224332	2.8842E-06
MMP1	Down	-2.77545	9.05794E-06
MTDH	Down	-2.0708454	0.000109479
MYC	Down	-2.3531933	6.84799E-06
MYO10	Down	-2.056971	0.000290178
NCAPG	Down	-3.466131	8.75417E-06
NCAPH	Down	-3.277411	3.37501E-07
NDC80	Down	-3.2344527	4.81342E-06
NDRG1	Down	-2.0377207	5.49884E-05
NDRG4	Up	2.0044627	5.06301E-05
NEK2	Down	-4.0638337	4.49023E-05
NMU	Down	-2.0142093	0.000157581
NR2F1	Up	2.3057506	2.93534E-05
NRP1	Up	2.192101	3.39564E-05
NUSAP1	Down	-4.121419	1.35285E-06
PBK	Down	-3.1371903	3.61418E-07
PCDH7	Up	3.6979644	3.16436E-05

Continued

Table S2 Continued

Target molecules in dataset	Regulation	Fold_change	*P*
PDGFRA	Up	3.8465552	0.000319564
PDIA5	Up	2.6322782	1.0041E-05
PGM3	Down	-2.1608644	4.79481E-05
PHGDH	Down	-2.5204659	2.53071E-06
PIK3R1	Up	3.5230632	2.80978E-06
PIK3R3	Up	4.3986635	7.12702E-07
PLK1	Down	-2.3198814	0.000164844
PODXL	Down	-2.613001	3.50673E-05
POLA1	Down	-2.0860755	2.76132E-06
POLE2	Down	-3.2632394	1.19058E-05
POSTN	Up	6.2268686	2.24186E-05
PPM1D	Up	2.0515914	0.002066009
PRC1	Down	-3.2625606	2.8762E-06
PRDM1	Up	2.4411225	3.35728E-05
PRIM1	Down	-3.0388367	2.39556E-05
PTCHD4	Up	2.135742	0.000167485
PTPN12	Down	-2.4150302	8.74551E-06
PTTG1	Down	-2.9523795	1.28204E-06
RACGAP1	Down	-2.4381306	4.61863E-05
RAD51	Down	-2.2705245	9.49069E-05
RAD51AP1	Down	-3.490721	6.10245E-05
RARRES3	Up	4.570907	6.67711E-07
RBL1	Down	-2.6507204	0.000324717
RBPJ	Down	-3.2799733	4.3E-05
RFC3	Down	-3.0650783	6.62013E-06
RFC4	Down	-2.6281676	3.20178E-06
RRM1	Down	-2.0249104	3.14287E-05
RRM2	Down	-2.6255248	7.52328E-07

Continued

Target molecules in dataset	Regulation	Fold_change	*P*
S100A2	Down	-2.0636077	2.59487E-06
SAT1	Up	2.3773856	1.38163E-06
SERPINB2	Down	-5.9836097	4.76691E-06
SERPINE1	Down	-2.776294	1.63496E-06
SERPINH1	Up	2.0949132	3.29312E-06
SESN1	Up	4.083665	1.25736E-07
SFRP2	Up	6.6169605	4.69607E-05
SGK1	Up	2.060982	1.9894E-05
SHISA5	Up	2.085424	3.4949E-06
SIDT2	Up	2.217279	0.000121514
SLC2A12	Up	2.389174	7.79558E-06
SMAD6	Up	2.2160177	1.37075E-06
SMAD7	Up	2.0452683	9.80671E-05
SMC4	Down	-2.0496526	1.14819E-05
SMURF2	Down	-2.0765357	1.56039E-05
SOD2	Up	2.3653908	5.75501E-08
SPATA18	Up	2.062645	0.000359106
SPC25	Down	-4.1329875	8.29516E-07
SPHK1	Down	-3.0104914	1.14056E-05
STMN1	Down	-2.428075	3.16331E-05
TDO2	Up	4.0305943	1.39229E-07
TFPI2	Down	-5.1304183	2.30218E-07
TGFB3	Up	7.6097136	5.65463E-05
TIGAR	Up	2.8314755	6.22895E-07
TMEM97	Down	-2.6563091	0.00019609
TNFAIP2	Up	3.5823321	1.6619E-06
TNFRSF10C	Up	3.342336	0.000138792
TNFRSF10D	Up	2.337418	6.22351E-06

Continued

Both table parts are headed: Downstream targets of p53 assessed *via* bioinformatic analysis after knocking down SNRPG

Table S2 Continued

Downstream targets of p53 assessed *via* bioinformatic analysis after knocking down SNRPG			
Target molecules in dataset	Regulation	Fold_change	*P*
TNFRSF9	Up	2.0453916	1.28021E-06
TNFSF10	Up	2.4190438	3.8299E-05
TOP2A	Down	-3.2117655	4.4514E-06
TP53I3	Up	3.2173285	3.44497E-05
TPD52L1	Up	5.191617	2.63324E-05
TPX2	Down	-3.0621989	3.22177E-07
TRIM22	Up	2.3660667	1.25788E-06
TSPAN6	Up	2.2814004	6.81893E-06
TTC28	Up	2.1429846	1.7392E-05
TTK	Down	-3.4548926	2.0953E-05
TUBB	Down	-2.0580049	1.89709E-06
TYMS	Down	-2.8719566	2.08657E-06
UBE2C	Down	-3.1527188	1.35925E-06
UBE2T	Down	-2.235396	8.42079E-07
UHRF1	Down	-2.9184792	2.20236E-05
USP14	Down	-3.0199425	3.90779E-05
VAMP4	Down	-2.2361658	5.1771E-07
VEGFA	Down	-2.4606857	5.41824E-07
VRK1	Down	-3.4682982	7.9567E-06
WDHD1	Down	-3.1272936	4.82984E-05
XAF1	Up	2.917754	0.000314485
ZMAT3	Up	2.7310157	1.09655E-06
ZNF175	Down	-2.184365	0.000152832

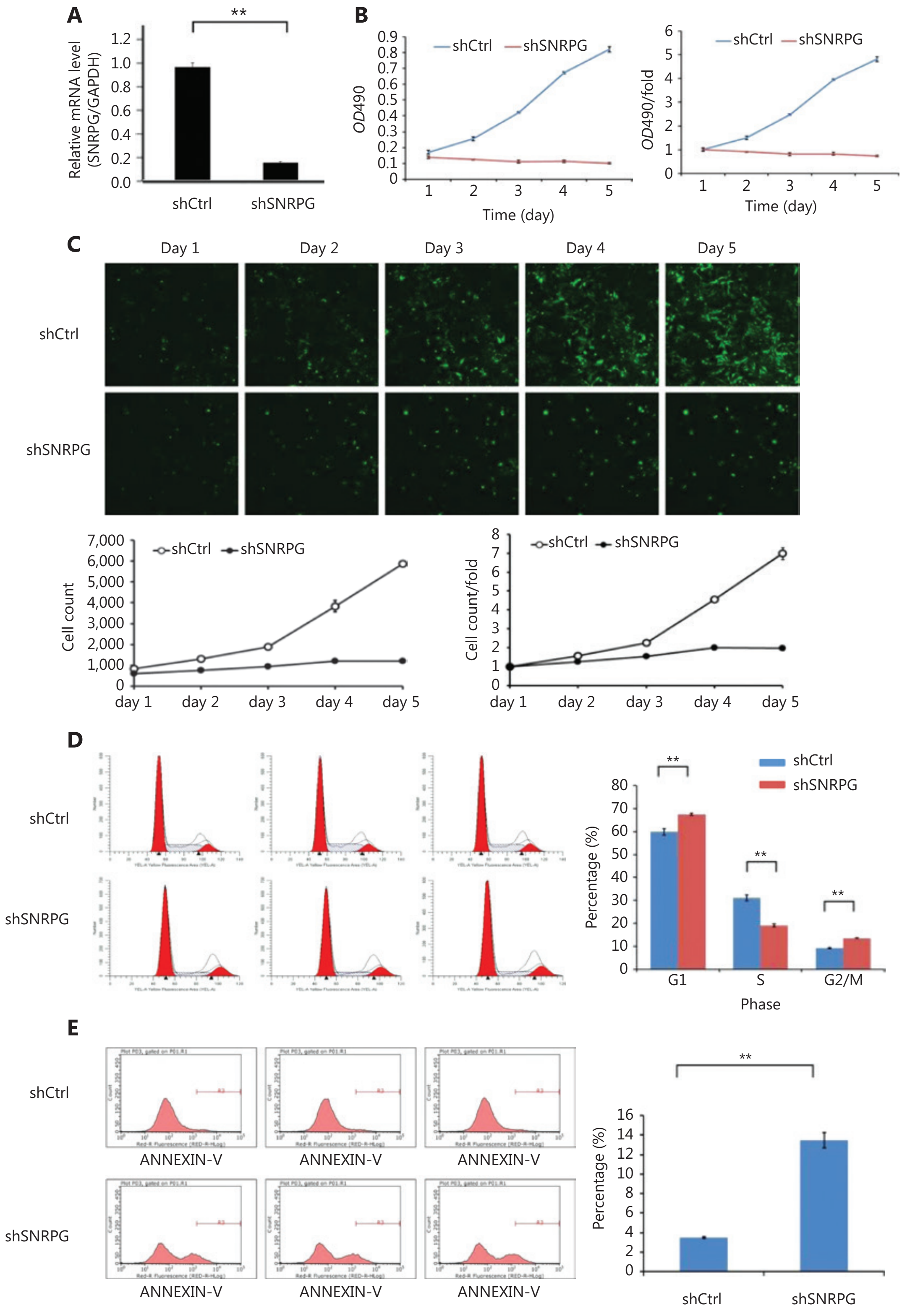
A
**
Relative mRNA level (SNRPG/GAPDH)
1.2
1.0
0.8
0.6
0.4
0.2
0.0
shCtrl
shSNRPG
B
OD490
shCtrl
shSNRPG
Time (day)
OD490/fold
C
Day 1
Day 2
Day 3
Day 4
Day 5
shCtrl
shSNRPG
Cell count
Cell count/fold
day 1
day 2
day 3
day 4
day 5
D
shCtrl
shSNRPG
Percentage (%)
G1
S
G2/M
Phase
E
ANNEXIN-V
Percentage (%)

Figure S1 Effects of knocking down *SNRPG* on cell proliferation in U251 cells. (A) *SNRPG* knockdown was performed using shRNAs in U251 cells. The downregulated expression of *SNRPG* by shSNRPG in U251 was further confirmed *via* RT-qPCR. (B) MTT assay showed that *SNRPG* knockdown significantly increased cell proliferation in U251 cells compared with the control cells. The *OD* values were measured at indicated days (mean ± SD, n = 3). (C) Celigo assay was performed to detect the cell viability over 5 days after transfection. The colony numbers of U251 cells transfected with shCtrl were evidently higher than those transfected with shSNRPG (mean ± SD, n = 3). (D–E) Effects of knocking down *SNRPG* on cell apoptosis and cell cycle progression in U251 cells. (D) Flow cytometric analysis was performed to further examine the effect of *SNRPG* on proliferation of GBM cells by altering cell cycle progression. Error bars, standard error. $^{**}P < 0.01$. (E) To further determine the physiological role of *SNRPG* in cells growth, U251 cells were transfected with shSNRPG. After 48 h, cell apoptosis were analyzed by flow cytometric analysis. Graphs show the mean percentages of three biological replicates. Error bars, standard error. $^{**}P < 0.01$.

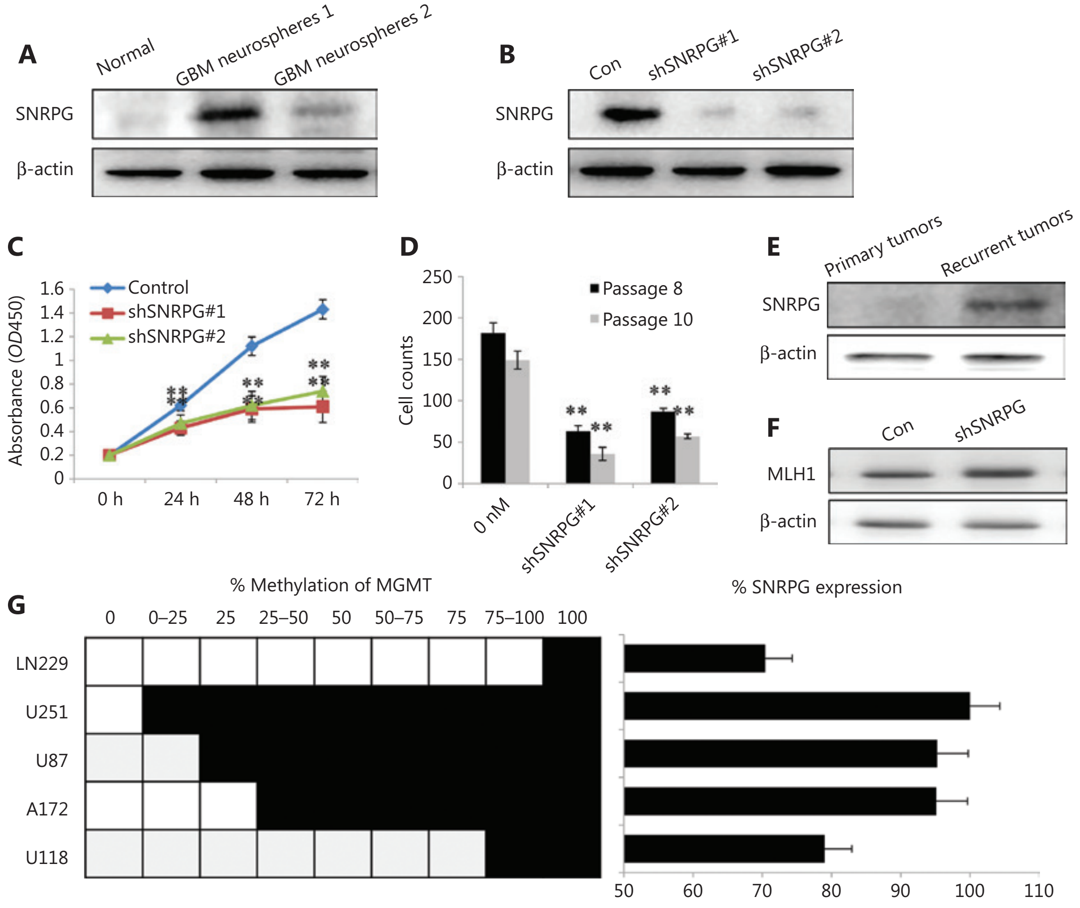

Figure S2 Expression of SNRPG in human GBM neurospheres and effect of silencing SNRPG on the proliferation and invasion of glioma cells. (A) The expression level of SNRPG in human normal brain tissue and GBM neurospheres were quantified by Western blot. (B) SNRPG protein levels in GBM neurospheres, as indicated, were determined by a Western blot analysis. β-actin was used as the internal control (C) CCK8 test to examine the influence of silencing SNRPG on the proliferation ability of GBM neurospheres. (D) Transwell invasion assay to test the influence of SNRPG inhibition on the invasion ability of GBM neurospheres. ($^{**}P < 0.01$). (E) The protein expression levels of SNRPG in samples of primary and recurrent GBM patients. n = 3. (F) Expression of mismatch repair (MMR) protein MLH1. MLH1 expression was significantly increased in shSNRPG compared to control group. (G) The possible correlation between SNRPG protein expression and MGMT promoter methylation percentage in GBM cell lines.

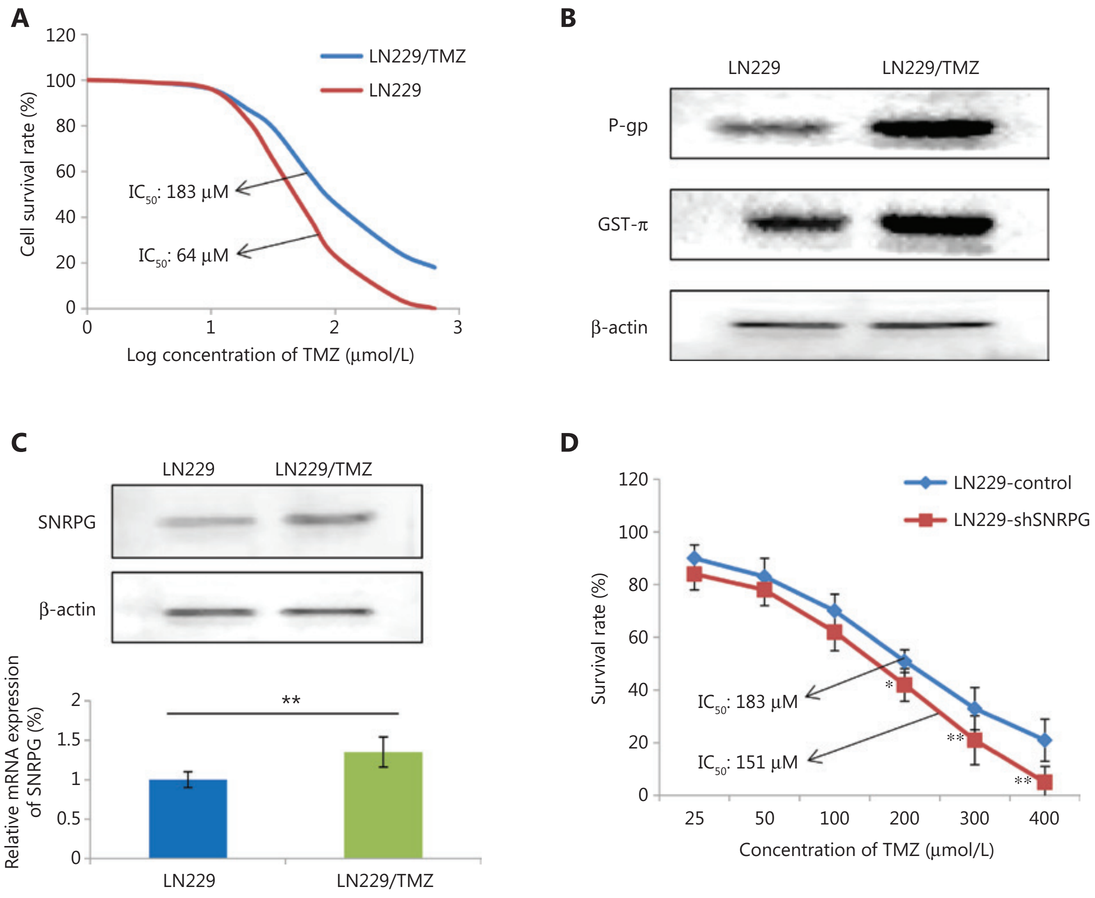

Figure S3 *SNRPG* suppression sensitizes human GBM cells to TMZ. (A) The MTT assay was used to determine the viability of LN229/TMZ (IC_{50}, 64 μM *vs.* 183 μM). (B) Western blot analysis of the drug resistance-related genes P-gp and GST-π in LN229/TMZ cells. (C) Western blot analysis (upper panel) and RT-qPCR analysis (lower panel) of *SNRPG* in LN229/TMZ cells. (D) The MTT assay was used to determine the cell viability after *SNRPG* knockdown. Downregulation of *SNRPG* significantly reduced the IC_{50} compared with that of the control group (151 μM *vs.* 183 μM; $^*P < 0.05$, $^{**}P < 0.01$).
